# CCN2–MAPK–Id-1 loop feedback amplification is involved in maintaining stemness in oxaliplatin-resistant hepatocellular carcinoma

**DOI:** 10.1007/s12072-019-09960-5

**Published:** 2019-06-27

**Authors:** Xia Liao, Yang Bu, Shanshan Jiang, Fan Chang, Fengan Jia, Xuelian Xiao, Ge Song, Mei Zhang, Pengbo Ning, Qingan Jia

**Affiliations:** 1 Department of Nutrition, First Affiliated Hospital of Xi’an Jiaotong University, Xi’an, 710061 China; 2 Department of Hepatobiliary Surgery, General Hospital, Ningxia Medical University, Yinchuan, 750001 China; 3 Institute of Hematological Research, Shaanxi Provincial People’s Hospital, Xi’an, 710068 China; 4 Metabolite Research Center, Shaanxi Institute of Microbiology, Xi’an, 710043 China; 5 Department of Hepatobiliary Surgery, First Affiliated Hospital of Xi’an Jiaotong University, 277 West Yanta Road, Xi’an, 710061 China; 6 School of Life Science and Technology, Xidian University, Xi’an, Shaanxi China

**Keywords:** Hepatocellular carcinoma, Oxaliplatin, CCN2, Id-1, MAPK signaling

## Abstract

**Background:**

Hepatocellular carcinoma (HCC) is the second leading cause of cancer death worldwide. Chemotherapy is an alternative treatment for advanced HCCs, but chemo-resistance prevents cancer therapies from achieving stable and complete responses. Understanding the underlying mechanisms in chemo-resistance is critical to improve the efficacy of HCC.

**Methods:**

The expression levels of Id-1 and CCN2 were detected in large cohorts of HCCs, and functional analyses of Id-1 and CCN2 were performed both in vitro and in vivo. cDNA microarrays were performed to evaluate the alterations of expression profiling of HCC cells with overexpression of CCN2. Finally, the role of downstream signaling of MAPK/Id-1 signaling pathway in oxaliplatin resistance were also explored.

**Results:**

The increased expression of Id-1 and CCN2 were closely related to oxaliplatin resistance in HCC. Upregulation of CCN2 and Id-1 was independently associated with shorter survival and increased recurrence in HCC patients, and significantly enhanced oxaliplatin resistance and promoted lung metastasis in vivo, whereas knock-down of their expression significantly reversed the chemo-resistance and inhibited HCC cell stemness. cDNA microarrays and PCR revealed that Id-1 and MAPK pathway were the downstream signaling of CCN2. CCN2 significantly enhanced oxaliplatin resistance by activating the MAPK/Id-1 signaling pathway, and Id-1 could upregulate CCN2 in a positive feedback manner.

**Conclusions:**

CCN2/MAPK/Id-1 loop feedback amplification is involved in oxaliplatin resistance, and the combination of oxaliplatin with inhibitor of CCN2 or MAPK signaling could provide a promising approach to ameliorating oxaliplatin resistance in HCC.

**Electronic supplementary material:**

The online version of this article (10.1007/s12072-019-09960-5) contains supplementary material, which is available to authorized users.

## Background

Hepatocellular carcinoma (HCC), which accounts for over 80% of primary liver cancers occurring worldwide, is the second leading cause of cancer death worldwide, with more than 50% of the total number of cases and deaths in China [1]. Despite progress in early detection and treatment of HCC, more than 70% of patients are at an advanced stage at diagnosis and are not suitable for curative therapies. Therefore, platinum-based local or systemic chemotherapy is an alternative treatment. Unfortunately, primary and acquired resistance to chemotherapy is common with HCC, which represents a major challenge in the treatment of advanced HCC [2]. Accordingly, the molecular characterization of chemo-resistant HCC is critical for further improving the efficacy of chemotherapy. In our previous cDNA microarray study of oxaliplatin-resistant HCCs, we have found significant upregulations of 267 genes including CCN2 and Id-1 (inhibitor of DNA binding protein-1) [3], while the accurate role and relationship of which in oxaliplatin resistance is still not yet clear in HCC.

The CCN family is a six-member family of cysteine-rich regulatory proteins in humans that share a multi-modular structure with an N-terminal secretory signal domain followed by four conserved domains [4]. Because they possess four functional domains, CCN proteins do not behave like traditional growth factors or cytokines and do not appear to have a unique receptor to which they bind with high affinity to induce signal transduction [5]. CCN2/Connective tissue growth factor (CTGF), one of the secreted factors upregulated in oxaliplatin-resistant HCC, is involved in proliferation, chemotaxis, adhesion, migration, and cell fate in different cell types and tissues [6]. However, evidences that suggest the role and mechanism of CCN2 in malignant tumors are still vague [7, 8].

Id-1 plays important roles in blocking cell differentiation and stimulating cell proliferation by mimicking the activities of other oncogenes, and inhibiting the tumor suppressor activities by targeting the proteins harboring the basic helix–loop–helix (HLH) motif [9]. Due to its role in cell differentiation, Id-1 has also been implicated in the biology of cancer stem cells [10]. Matsuda et al. [11] reported that increased Id-1 expression in HCC plays an important role in hepatocarcinogenesis and serves as a useful marker for risk prediction of occurrence. Sharma et al. [12] found that Id-1 has a tumor promotion role in the metabolic reprogramming of cancer cells including aerobic glycolysis and glutaminolysis. Further, the low postnatal expression of Id-1 and its high expression in cancer stem cells mark them as attractive targets for anti-cancer therapy. However, the role of Id-1 in HCC and the regulatory mechanisms it shares with CCN2 remain unclear, especially in HCC with oxaliplatin resistance.

In the present study, we determined that increased Id-1 and CCN2 expression were closely correlated with oxaliplatin resistance in HCC, verified that malignancy and poor prognosis were associated with Id-1 in human HCC, and explored the negative roles of Id-1 and the regulatory mechanisms of CCN2 in HCC. Finally, we demonstrated enhanced oxaliplatin resistance could be reversed by CCN2–MAPK–Id-1 signaling loop inhibition.

## Methods

### Patients and follow-up

A total of 268 patients who underwent curative liver resection for HCC between January 2004 and December 2006 at the Zhongshan Hospital Fudan University were enrolled in this study. Among which, the training set contained 64 cases (including HCC tissues and the paired non-tumor liver tissues from 48 patients were used for real-time PCR and that from 16 patients for Western blot), another set of 184 patients was used for validation (the validation set). Curative resection was defined as the complete resection of tumor nodules, leaving the tumor margins free of cancer upon histologic examination. The histopathologic diagnosis was confirmed by two independent experienced pathologists. Patients were followed after surgical treatment until December 2013, and the median follow-up was 63 months (range 0–110 months). Details of the follow-up procedures were previously described [13]. The clinicopathologic characteristics of HCC patients in the validation set were provided in Supplementary Tables 1 and 2.

### Animal model and treatment procedures

MHCC97H-CCN2-sh cells, Hep3B-CCN2 cells, MHCC97H-Id1-sh cells, MHCC97H-Id1 cells, MHCC97H mock cells, and their associated control cells were implanted subcutaneously into the upper left flank region of mice to establish subcutaneous xenografts. The synergistic effects of the combination therapy of oxaliplatin and sorafenib were evaluated. Twenty-four nude mice bearing subcutaneous xenografts were randomly divided into the control, oxaliplatin, sorafenib, and oxaliplatin + sorafenib groups (*n* = 6 per group). The treatments included a tail vein injection of oxaliplatin (10 mg/kg/week), a 0.2 mL oral dose of sorafenib (30 mg/kg/week). Tumor weights were evaluated in 4 weeks after the treatments. The subcutaneous implantation models were also established in 12 C57 mice using Hep1-6 cell, to carry out the same above evaluations. Intraperitoneal injection of pentobarbital (5 mg/kg) combined with cervical spondylolisthesis was used for the killing of mice after the study.

### cDNA microarray analysis

cDNA microarrays were performed using the Human OneArray^®^ (Phalanx Biotech Group, San Diego, CA) to evaluate the alterations of expression profiling. Total RNA was extracted from Hep3B-CCN2 and Hep3B-vector cells and the isolations and microarray analyses were performed in triplicate according to the manufacturer’s instructions. All data were uploaded to the Gene Expression Omnibus (GEO accession number 124529).

### Vector construction, transfection, and lentivirus transduction

The human full-length Id-1 (NM_002165) and CCN2 (NM_001901.2) cDNA were obtained from GeneCards (Shanghai, China) and cloned into the pCDH lentiviral expression vector (System Biosciences, CA, USA). Using the In-Fusion^®^ HD Cloning Kit (Takara, Tokyo, Japan), the amplified fragment was inserted into the pCDH plasmid (between XbaI and EcoRI sites). Lentiviral shRNA expression plasmids PLVT, PLKO.1, and the target sequences were listed in Supplementary Table 7.

### Statistical analysis

Statistical analyses were performed using SPSS 15.0 for Windows (SPSS, Inc., Chicago, IL). A *p* value < 0.05 was considered statistically significant.

## Results

### Increased expression levels of Id-1 and CCN2 were closely related to oxaliplatin resistance in HCC

**Fig. 1:**
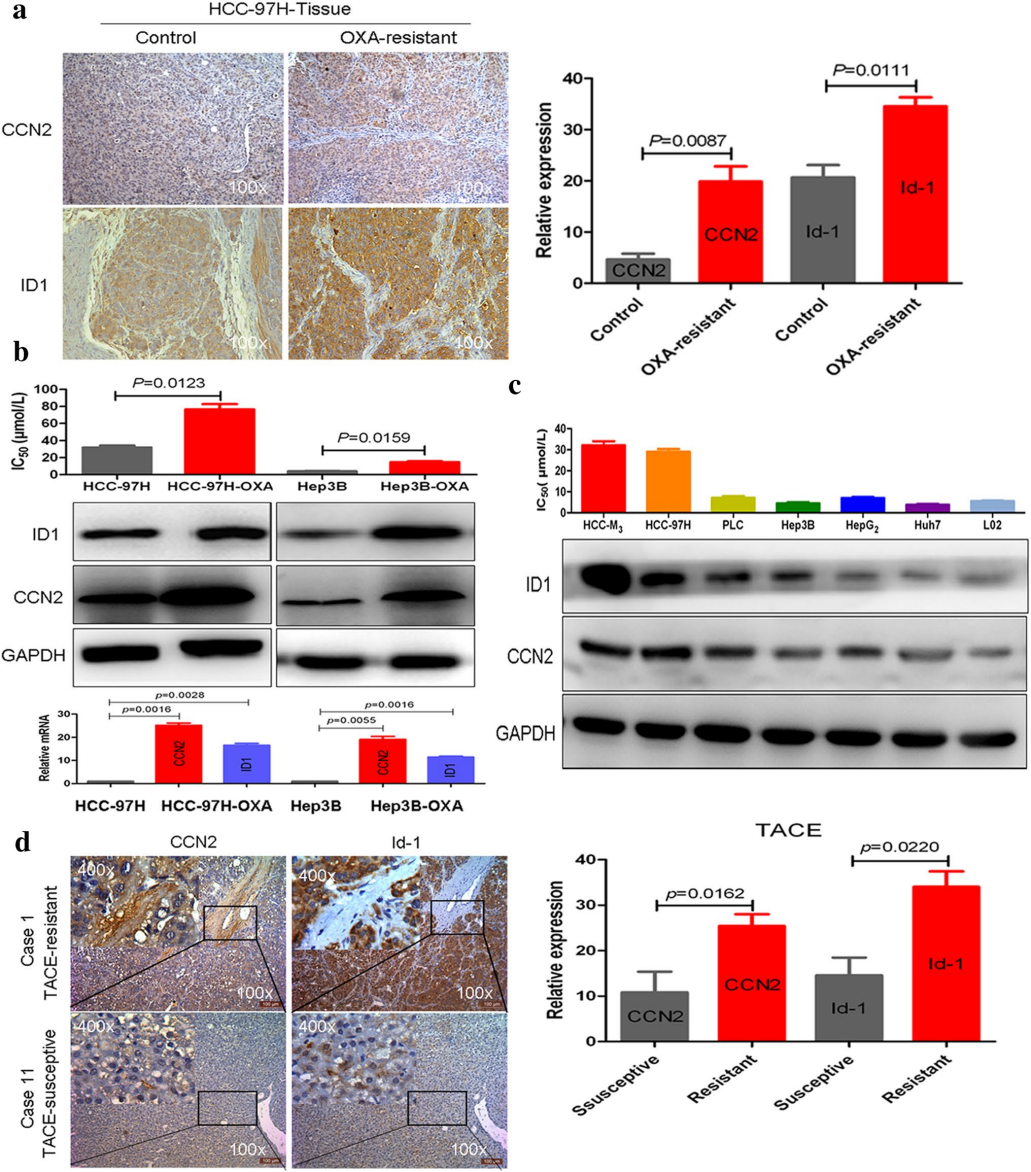
Increased Id-1 and CCN2 expressions were closely related to oxaliplatin resistance in HCC. **a** Increased CCN2 and Id-1 expression was verified in oxaliplatin-resistant subcutaneous tumors by immunohistochemistry. **b** Increased Id-1 and CCN2 expressions were shown in oxaliplatin-resistant MHCC97H-OXA and Hep3B-OXA cells by Western blotting and real-time PCR. **c** Id-1 and CCN2 expressions were explored in seven HCC cell lines with different oxaliplatin resistance malignancy phenotypes. **d** Increased Id-1 and CCN2 expressions were also shown in TACE-resistant patients

The increased expression levels of CCN2 and Id-1 in the subcutaneous oxaliplatin-resistant tumors were confirmed by immunohistochemical staining (Fig. 1a). Significantly increased expression of vimentin, CD44, aldehyde dehydrogenase 1 (ALDH1), and epithelial cell adhesion molecule (EpCAM), which are related to epithelial–mesenchymal transition (EMT) and stemness, were also upregulated in subcutaneous oxaliplatin-resistant tumors (Supplementary Fig. 1). The expression levels of Id-1 and CCN2 in the oxaliplatin-resistant HCC cell lines, MHCC97H-OXA and Hep3B-OXA, were demonstrated to be significantly increased by Western blot and real-time PCR (Fig. 1b). The expression levels of Id-1 and CCN2 were investigated in seven HCC cell lines with different malignancy phenotypes of oxaliplatin resistance (IC_50_: HCC-M3, 32.10 ± 3.28; HCC-97H, 28.98 ± 2.37; PLC, 7.10 ± 1.49; Hep3B, 4.52 ± 0.88; HepG2, 7.01 ± 0.75; Huh7, 3.86 ± 0.58; L02, 5.78 ± 0.32). Both the Id-1 and CCN2 levels were significantly increased in HCC cell lines with high metastatic potentials and strong resistance to oxaliplatin (HCC-LM3 and HCC-97H), whereas relatively lower CCN2 and Id-1 levels were detected in HCC cell lines with low metastatic potential and weak oxaliplatin resistance (PLC, HepG_2_, Huh7, and Hep3B) and in the human liver cell line (L-02; Fig. 1c). Both Id-1 and CCN2 levels were also obviously increased in HCC patients who were resistant to transcatheter arterial chemoembolization (TACE) compared with those TACE-susceptible patients (Fig. 1d; Supplementary Fig. 2).

### Increased Id-1 and CCN2 expression trends are positively associated with HCC malignancy and poor prognosis

**Fig. 2:**
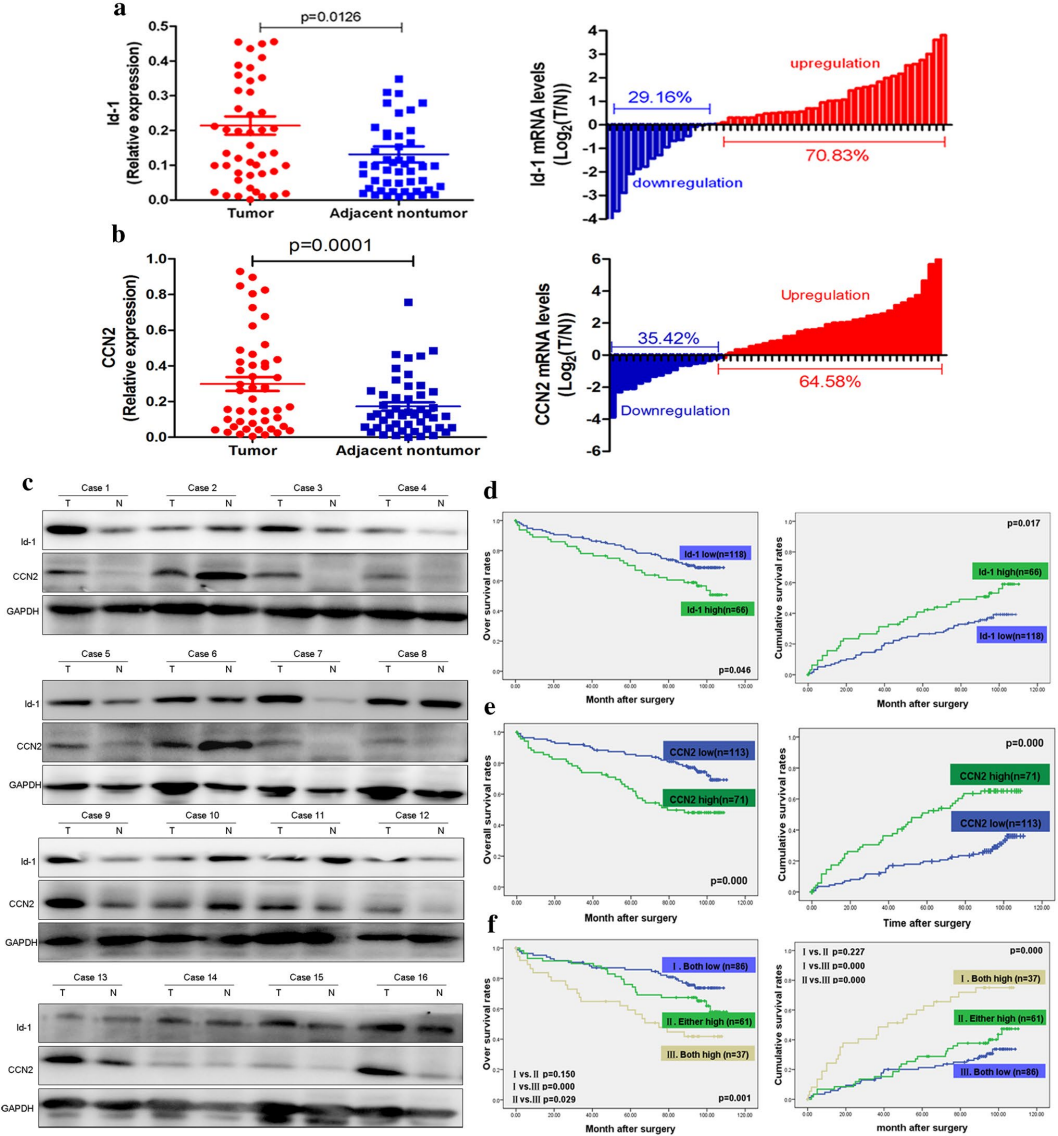
Id-1 and CCN2 expressions in HCC samples were higher compared with non-tumor liver tissues and were positively associated with poor prognosis. **a**, **b** Increased Id-1 and CCN2 expression were observed in HCC samples compared with non-tumor tissues. **c** A positive correlation between the expression levels of Id-1 and CCN2 was demonstrated in proteins level of 16 randomly selected HCC samples. **d**, **e** The patients with high Id-1 or CCN2 expression had significantly lower OS and higher CRR. **f** The patients with both high Id-1 and CCN2 expression had the lowest OS and highest CRR than low expression levels of both CCN2 and Id-1, or either low expression levels of CCN2 or Id-1

We detected the mRNA expression levels of Id-1 and CCN2 in 48 HCCs and their adjacent non-tumor liver tissues, and found increased Id-1 expression in 70.83% (34/48), and increased CCN2 expression in 64.58 (31/48) of HCC tissues compared with the corresponding non-tumor liver tissues (Fig. 2a, b). These results were validated in proteins level of 16 randomly selected HCC samples, and a positive correlation between the expression levels of Id-1 and CCN2 was demonstrated (Fig. 2c).

To illustrate the clinical role of Id-1 and CCN2 in HCC, 184 patients in the validation cohort were sorted according to the Id-1 and CCN2 expression levels (Supplementary Fig. 3A, B). The patients in the Id-1^high^ group had significantly lower overall survival (OS) and higher cumulative recurrence rates (CCR) compared with those in the Id-1^low^ group (Fig. 2d). The patients in the CCN2^high^ group also had significantly lower OS and higher CCR compared with those in the CCN2^low^ group (Fig. 2e). We then classified the patients into three subgroups according to CCN2 and Id-1 expression levels. Group I had low expression levels of both CCN2 and Id-1, group II had high expression of either CCN2 or Id-1, and Group III had high expression of both CCN2 and Id-1. The patients in group I had the best prognosis, their OS rate was significantly higher than that of the patients in groups II and III, and their CCR was significantly lower (Fig. 2f).

Cox regression analysis revealed that increased Id-1 expression was only significantly correlated with tumor differentiation. No significant association was found between Id-1 expression and the other clinico-pathological characteristics including age, gender, hepatitis B virus (HBV) surface antigen (HBsAg) status, cirrhosis, serum alpha-fetoprotein (AFP) and alanine aminotransferase (ALT) levels, tumor dimension, tumor number, vascular invasion, or tumor encapsulation (Supplementary Table 1). Cox regression analysis also revealed that increased CCN2 expression was significantly correlated with tumor number and tumor differentiation. However, no significant association was found between CCN2 expression level and the other clinical and pathological characteristics (Supplementary Table 2).

A univariate analysis revealed that tumor size, tumor number, vascular invasion, Id-1 and CCN2 expression were significantly associated with the post-operative OS and CCR of HCC patients. However, no prognostic significance was found for the other characteristics including age, gender, HBsAg, HCV-Ab, liver cirrhosis, AFP, ALT, tumor encapsulation or differentiation (Supplementary Table 3). The multivariate Cox proportional hazards model revealed that tumor size, number of tumor nodules, and Id-1 and CCN2 expression were also independent prognostic indicators for OS and CCR of HCC patients; however, vascular invasion was an independent prognostic indicator for OS in HCC patients (Supplementary Table 4).

To confirm the prognostic value of Id-1 and CCN2 for HCC, we detected its protein levels in frozen tissue samples from HCC patients by immunoblotting, and found a significantly increased Id-1 and CCN2 protein levels in HCC tissues with early recurrence compared with the others. In addition, increased Id-1 and CCN2 expression was found to be associated with poor differentiation of HCC (Supplementary Fig. 4).

### Upregulation of Id-1 and CCN2 are related to enhanced stemness of HCC cells

**Fig. 3:**
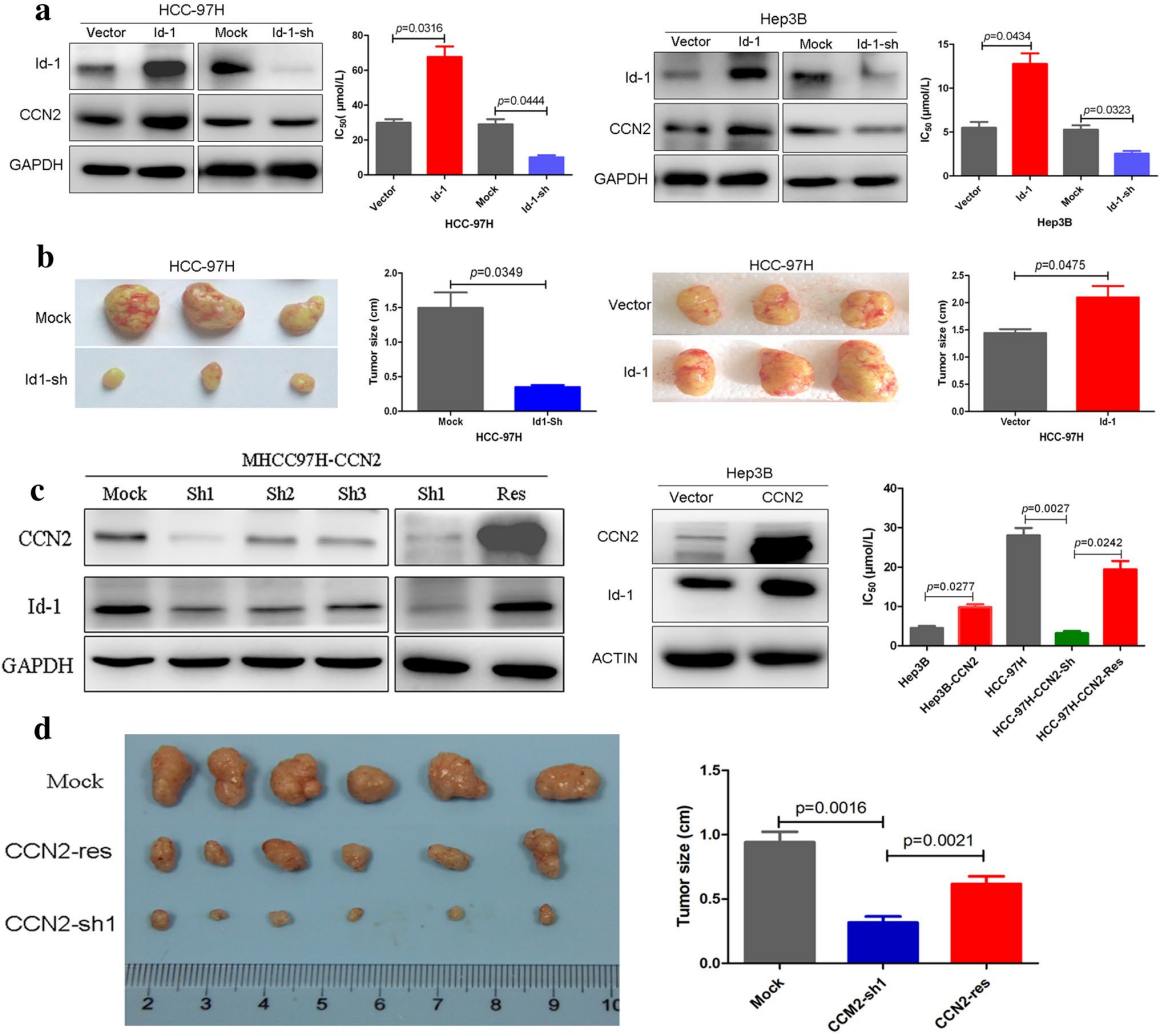
Increased expression of Id-1 and CCN2 was related to oxaliplatin resistance and enhanced proliferative capacity in HCC. **a** Id-1 was stably overexpressed and silenced in HCC cell lines following the partly alteration of CCN2, and the upregulation of Id-1 enhanced oxaliplatin resistance but was reversed after Id-1 silencing. **b** HCC-97H cells overexpressing Id-1 exhibited enhanced subcutaneous tumor growth capacity that was reversed after Id-1 silencing. **c** CCN2-sh1 was found to exert the most efficient interference of CCN2 following the significant alteration of Id-1, and after rescue of CCN2 expression in HCC-97H, or overexpression of CCN2 in Hep3B, the expression of Id-1 were upregulated. **d** The in vivo subcutaneous tumor growth capacity of MHCC-97H cells transfected with CCN2 shRNA in nude mouse models was significantly diminished, whereas it is significantly enhanced when the CCN2 was rescued

To evaluate the exact function of Id-1, we stably overexpressed and silenced Id-1 expression in HCC cells. The upregulation of Id-1 significantly enhanced oxaliplatin resistance which could be reversed after Id-1 expression was silenced (Fig. 3a). The migration, invasion, and sphere formation abilities of MHCC-97H cells were also significantly enhanced following overexpression of Id-1, and were inhibited after Id-1 silencing (Supplementary Fig. 5). The in vivo subcutaneous tumor growth capacity of MHCC-97H cells transfected with Id-1 shRNA in nude mouse models was significantly diminished, whereas it is significantly enhanced when the Id-1 was upregulated through lentiviral transfection in MHCC-97H cells (Fig. 3b). Moreover, all of the mice with subcutaneous implantation of MHCC-97H cells transfected with Id-1 expression vector exhibited pulmonary metastasis, with an average of more than five metastatic nodules per lung, whereas none of the mice subcutaneous implantation of MHCC-97H cells transfected with control vector was found to have lung metastases (Supplementary Fig. 6). Alterations of the key stemness-related molecules in subcutaneous tumor tissues were investigated by immunohistochemistry. In the Id-1-sh group, the expression levels of CD44, EpCAM, Osteopontin (OPN) and ALDH were significantly downregulated compared with the Mock group, and were significantly upregulated after Id-1 expression was overexpressed (Supplementary Fig. 7). These indicate that Id-1 is significantly involved in oxaliplatin resistance and stemness in HCC.

To evaluate the role of CCN2 in HCC, we stably silenced and restored CCN2 expression in HCC cells. Among of the three CCN2-shRNA, CCN2-sh1 was found to exert the most efficient interference of CCN2 compared with MHCC-97H-mock cells, and restoration of CCN2 expression could rescue the altered expression of CCN2 (Fig. 3c; Supplementary Fig. 8). The downregulation of CCN2 significantly inhibited the oxaliplatin resistance which could be reversed when CCN2 expression was restored. The downregulation of CCN2 expression significantly impaired the invasiveness, migration, proliferation and sphere formation abilities compared with the controls. Furthermore, after rescuing CCN2 expression, the impaired abilities were significantly restored (Supplementary Fig. 9). The in vivo subcutaneous tumor growth capacity of MHCC-97H cells transfected with CCN2 shRNA in nude mouse models was significantly diminished, whereas it is significantly enhanced when the CCN2 was rescued (Fig. 3d).

### Overexpression of CCN2 induced significant changes in gene expression profiles of HCC cells

**Fig. 4:**
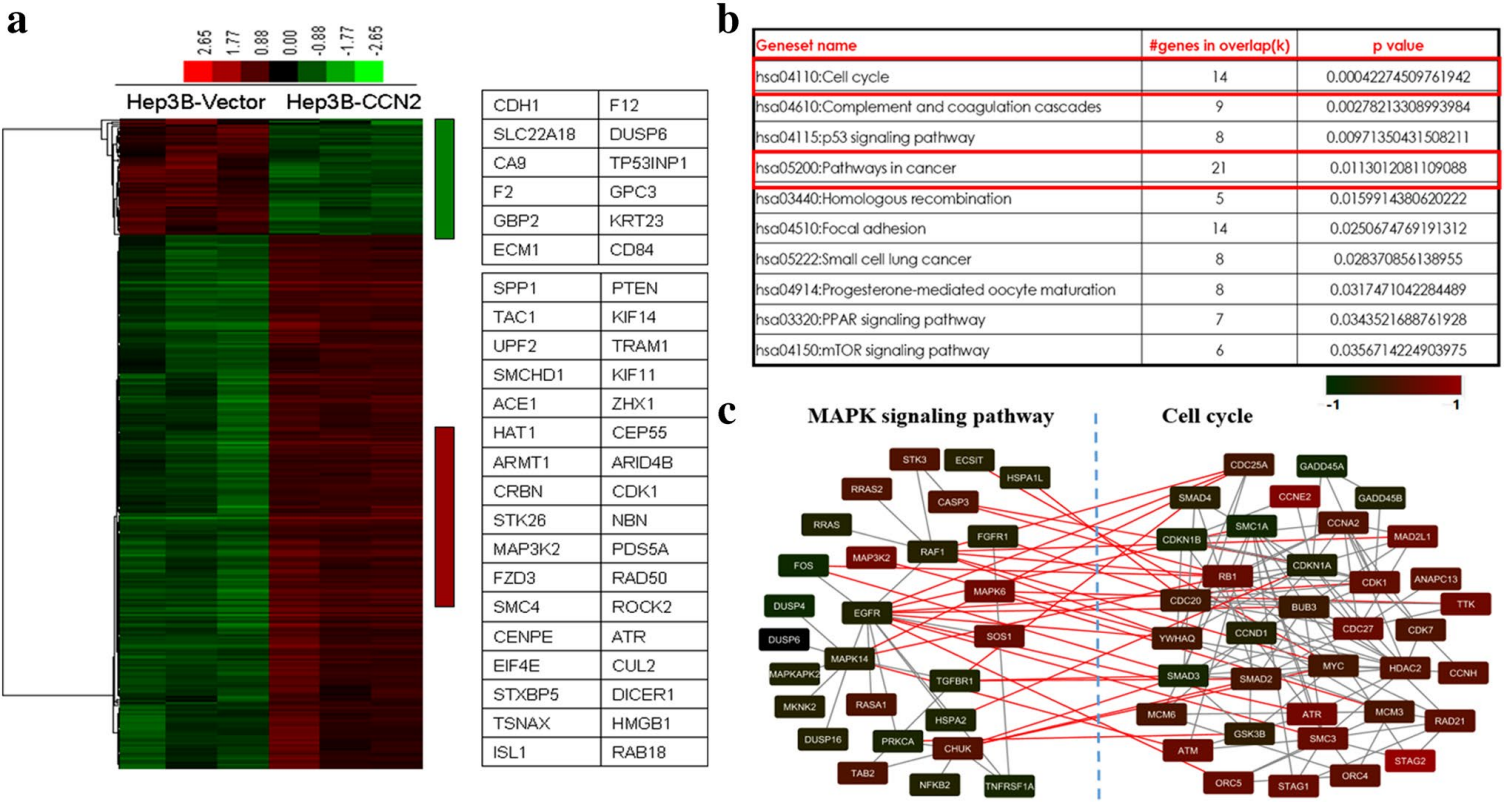
Gene expression profiles were significantly altered after overexpression of CCN2. **a** The expression of 32,679 genes was compared between Hep3B-vector and Hep3B-CCN2, which revealed 581 upregulated and 138 downregulated genes. **b** The top 10 significantly enriched GO terms were selected and we determined that cell cycle was a significantly altered group with 14 significantly modulated genes. **c** A close relationship between MAPK signaling and cell cycle was noted and the inferred regulatory network was built

To investigate the potential contribution of CCN2 to the stemness of HCC cells, we compared the gene expression profiles (32,679 genes) of Hep3B-CCN2 and Hep3B-vector using cDNA microarrays. The > twofold differences in expression levels were found in 768 genes, including 581 upregulated and 167 downregulated genes, between Hep3B-CCN2 and Hep3B-vector cells (Fig. 4a). Differentially expressed genes in HCC with CCN2 overexpression were evaluated by Gene Ontology (GO) enrichment in the Gene Set Enrichment Analysis. The top ten significantly enriched GO terms were selected (Fig. 4b), and 14 genes related to cell cycle including *CDK1*, *ROCK2*, *CCNE2*, *CCNT2*, *TTK*, *PTEN*, *MAP3K2*, *MAPK6*, *SOS1*, and *RASA1* were determined to be significantly changed. Further evaluation of signaling pathway using the Kyoto Encyclopedia of Genes and Genomes (KEGG) database revealed that the MAPK signaling pathway was significantly enriched, which indicates a common regulatory network between MAPK signaling and cell cycle (Fig. 4c).

Interestingly, *spp1*, which encodes OPN, a secreted phosphoprotein that plays a crucial role in HCC metastasis [14–17], was also significantly upregulated in HCC with CCN2 overexpression, whereas *CDH1*, which encodes E-cadherin and mediates the epithelial phenotype in tumor cells [18], was significantly downregulated. Many other significantly altered genes that play important roles in tumor progression were also identified to be closely related to CCN2 (all data were uploaded into GEO).

### CCN2 significantly activates the MAPK/Erk/Id-1 signaling pathway and Id-1-positive feedback amplified the expression of CCN2

**Fig. 5:**
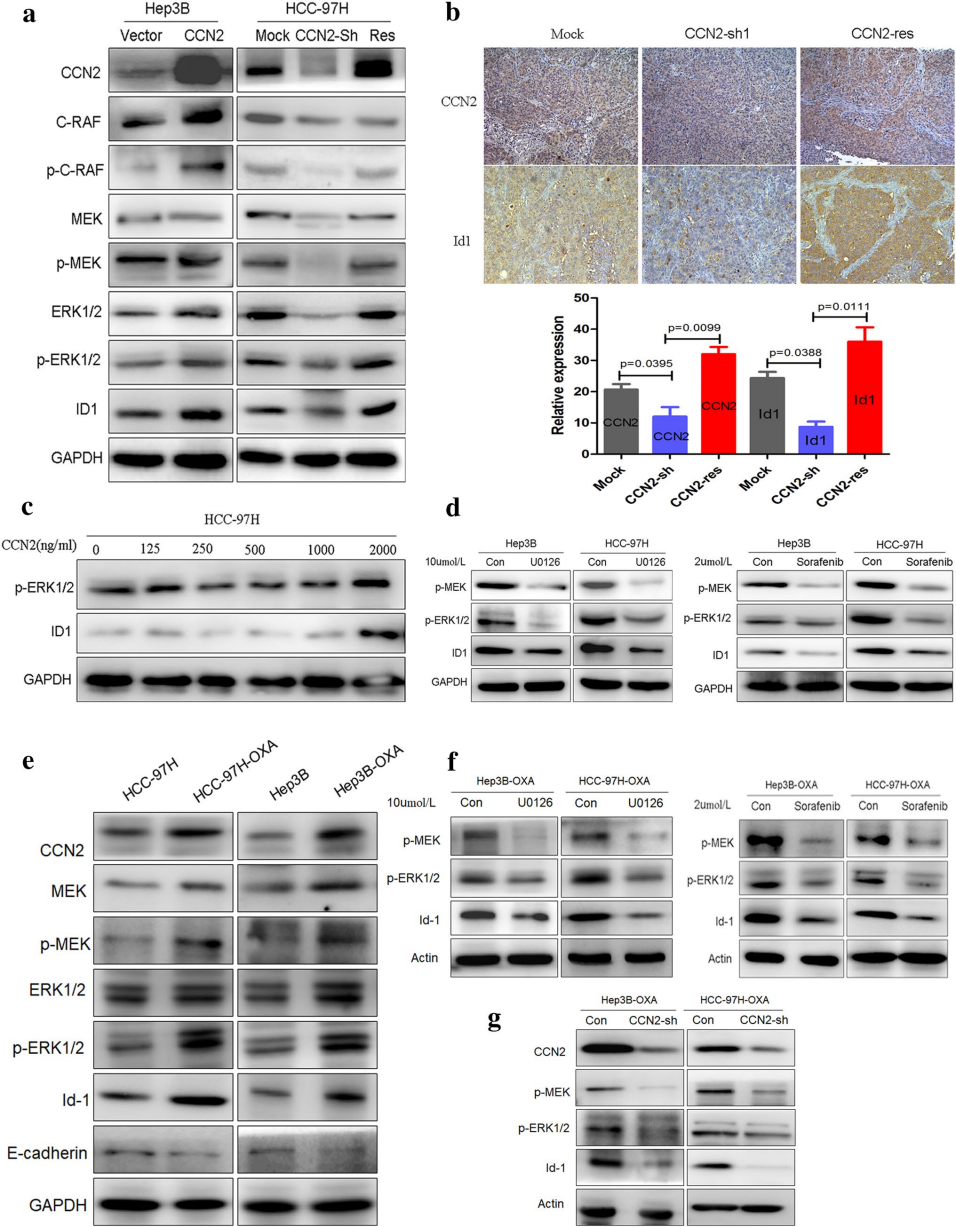
CCN2 significantly activates the MAPK/Erk signaling pathway and upregulates Id-1 expression. **a** The activation of the MAPK/Erk signaling cascade and the upregulation of Id-1 were positively related to CCN2. **b** The upregulation of Id-1 was positively related to CCN2 in subcutaneous tumor tissue. **c** Recombinant CCN2 activated MAPK/Erk signaling and upregulated Id-1 expression in a dose-dependent manner. **d** Sorafenib and U0126 significantly inhibited MAPK/Erk signaling and downregulated Id-1 expression. **e** Activation of the MAPK/Erk signaling cascade and upregulation of Id-1 were confirmed in oxaliplatin-resistant MHCC97H-OXA and Hep3B-OXA cells. **f**, **g** MAPK/Erk signaling was impaired and Id-1 expression was downregulated following sorafenib, U0126 treatment, or silencing endogenous CCN2 in oxaliplatin-treated HCC cells

To investigate the effect of CCN2 on Id-1 expression and the associated MAPK/Erk signaling, we overexpressed CCN2 in Hep3B cells, and found that CCN2 activated the MAPK/Erk signaling cascade with concomitant upregulation and phosphorylation of p-C-RAF, p-MEK, and p-Erk1/2, and ultimately upregulated Id-1 levels. On the other hand, when the CCN2 expression in HCC-97H cells was silenced using specific shRNA, the MAPK/Erk signaling and Id-1 expression were obviously inhibited. Moreover, after rescuing CCN2 expression, the impaired MAPK/Erk signaling and Id-1 expression were reversed (Fig. 5a). In the HCC tissues from subcutaneous implantation model of mice, the knock-down of CCN2 was significantly associated with a decreased protein level of Id-1 detected by immunohistochemical staining, and the impaired Id-1 expression were reversed after rescuing CCN2 expression (Fig. 5b). Taking together, these suggest that CCN2 plays important roles in the regulation of MAPK/Erk signaling cascade and Id-1 expression. To validate this mechanism, we treated HCC cells with recombinant CCN2 in a dose-dependent manner (0–2000 ng/m) and found that the p-Erk and Id-1 expression levels were significantly increased (Fig. 5c). And, sorafenib (2 μmol/L) or the MEK1/2 inhibitor U0126 (10 μmol/L) treatment induced a significant inhibition on MAPK/Erk signaling and the downregulation of Id-1 (Fig. 5d).

In the case of construction of oxaliplatin-resistant HCC cell lines, firstly, we treated the oxaliplatin-resistant MHCC-97H cells with different concentrations of oxaliplatin, and found that the expression levels of CCN2, Id-1, LRP6, p-Erk, and E-cadherin varied in a dose-dependent manner (Supplementary Fig. 10A). And, the variations in CCN2, Id-1, and p-Erk expression levels also occurred in a time-dependent pattern with a significant difference in the maximum time point during the oxaliplatin treatment (Supplementary Fig. 10B).

We re-analyzed the difference between oxaliplatin-resistant and wild-type HCC, and the activation of the MAPK/Erk signaling cascade was demonstrated to be concomitant with the upregulation and phosphorylation of p-C-RAF, p-MEK, and p-Erk1/2, which eventually led to the upregulation of Id-1 (Fig. 5e). When sorafenib or U0126 was added to the oxaliplatin-resistant HCC cells, the MAPK/Erk signaling pathway was impaired and Id-1 expression was downregulated (Fig. 5f). On the other hand, when the CCN2 expression in oxaliplatin-resistant HCC cells was silenced using specific shRNA, the MAPK/Erk signaling and Id-1 expression were obviously inhibited (Fig. 5g). Furthermore, Id-1 overexpression or interference induced an obvious variation of CCN2 protein level in the same trend in HCC cells (Fig. 3a).

### Oxaliplatin combined with CCN2/MAPK/Erk signaling inhibition results in improved treatment effects in HCC

**Fig. 6:**
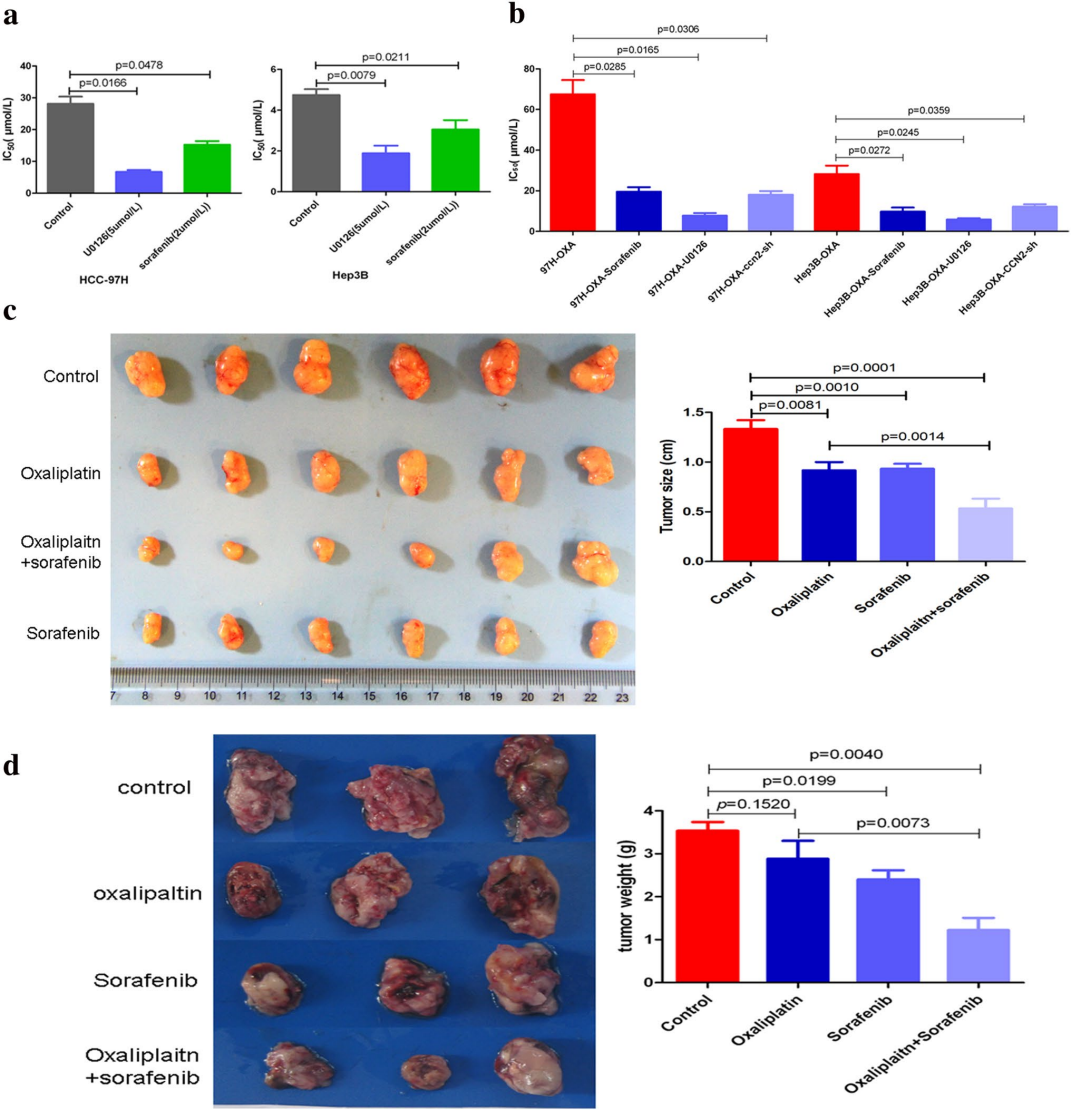
Oxaliplatin combined with CCN2/MAPK/Erk signaling inhibition results in improved treatment effects in HCC. **a** Sorafenib or U0126 combined with oxaliplatin reduced the IC_50_ of HCC cells to oxaliplatin. **b** The IC_50_ of HCC cells to oxaliplatin was decreased in oxaliplatin-resistant HCC cells after silencing endogenous CCN2 or combining oxaliplatin with sorafenib or U0126. **c** The combined treatment with sorafenib and oxaliplatin resulted in a significant inhibitory effect in subcutaneous xenograft models established in nude mice using MHCC-97H cells. **d** The combined therapy also showed a more significant antitumor effect on HCC in subcutaneous xenograft models established in C57BL/6 mice using Hep1-6 cells

As mentioned above, sorafenib and the MEK1/2 inhibitor U0126 could significantly inhibit MAPK/Erk signaling and downregulate the expression of Id-1. We treated MHCC-97H and Hep3B cells with the combination of sorafenib or U0126 with oxaliplatin to investigate their possibilities in reversing oxaliplatin resistance of HCC cells, and found that the combination of sorafenib or U0126 significantly decreases the IC_50_ of HCC cells to oxaliplatin (Fig. 6a). The synergistic effect of sorafenib, U0126, or CCN2 silencing in oxaliplatin-resistant HCC cells also resulted in inducing a significantly decreased IC_50_ of HCC cells to oxaliplatin (Fig. 6b).

Furthermore, in the subcutaneous xenograft models established in nude mice using MHCC-97H cells, the combination of sorafenib with oxaliplatin induced a more significant inhibitory effect on in vivo tumor growth compared with the oxaliplatin alone (Fig. 6c). In the subcutaneous xenograft models of C57BL/6 mice established with Hep1-6 cells, oxaliplatin did not exhibit an inhibitory effect on tumor growth, but when in combination with sorafenib, oxaliplatin exhibited a significant inhibitory effect on in vivo growth of HCC (Fig. 6d).

## Discussion

**Fig. 7:**
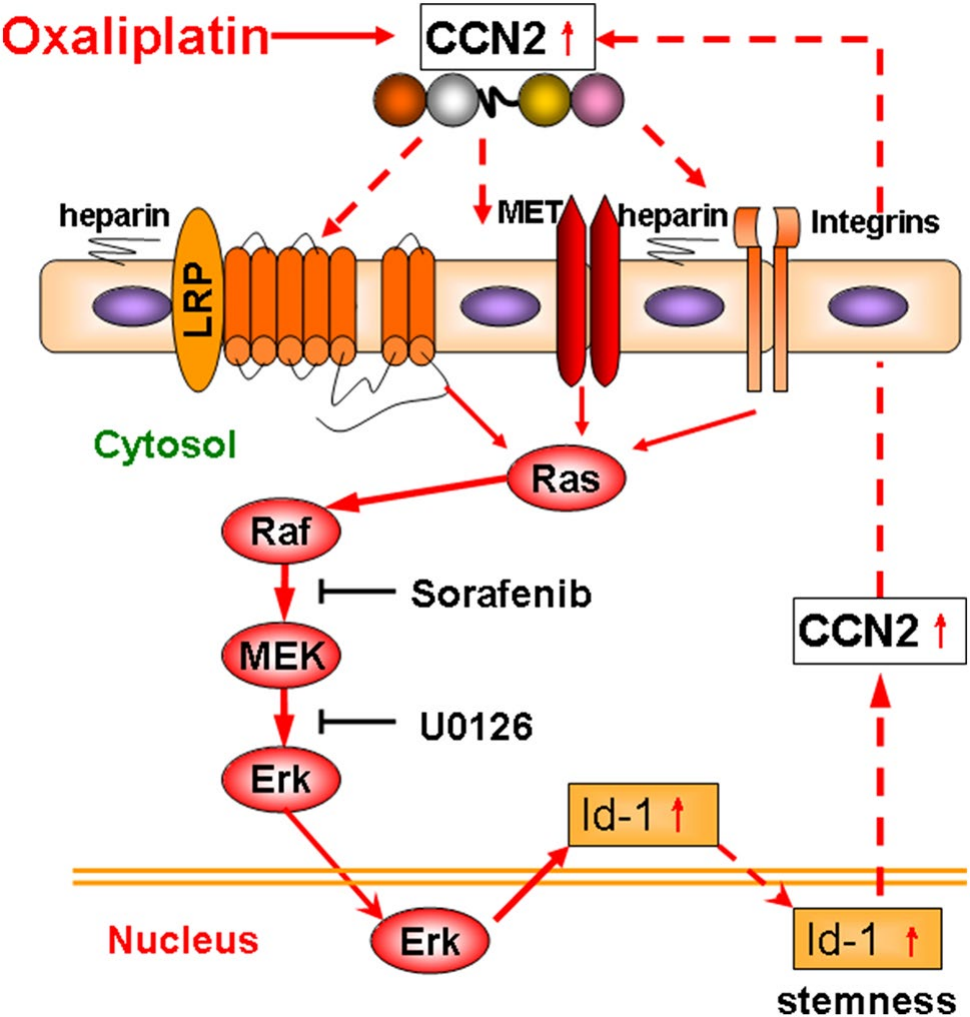
The upregulation of CCN2 are closely related to oxaliplatin treatment in HCC, and CCN2 interacting with many cell membrane receptors such as LRP6, Met, integrins, etc., significantly enhanced oxaliplatin resistance by activating the MAPK/Id-1 signaling pathway. Id-1 upregulate CCN2 in a positive feedback manner, and sorafenib or U0126 could significantly inhibit Id-1 expression by blocking the CCN2–MAPK–Id-1 loop

For HCC patients diagnosed at early stages, potentially curative treatments including radiofrequency ablation, resection, and liver transplantation are available. Unfortunately, most patients with advanced HCC are not eligible for curative therapy, and must rely on local and systemic therapy [19, 20]. The current standard treatment for advanced hepatocellular carcinoma (HCC) is sorafenib. Although sorafenib is effective in the early stage, the general survival rate is not satisfied and other treatment options are still needed [21, 22]. Hence, platinum-based chemotherapy for advanced HCC patients has been revisited extensively in both Western and Eastern patient populations [23]. However, a phase II study of single-agent oxaliplatin in patients with unresectable, metastatic, or recurrent HCC failed to meet the a priori therapeutic criterion [24]. In recent years, several studies have investigated combination or sequential methods to reduce the toxicity and enhance the sensitivity of chemotherapy. Zhu et al. [25] evaluated GEMOX plus bevacizumab in a randomized phase II study and found that the combination demonstrated manageable toxicity and encouraging tumor responses. In results of a phase I trial in colorectal cancer patients, continuous oral sorafenib was safely combined with oxaliplatin, and showed significantly antitumor activity [26]. In HCC, exploratory analyses are underway to refine the selection of patients likely to derive the most benefit from the oxaliplatin-based combination therapy (Fig. 7).

Previously, our research group studied various HCC therapies, including curative and palliative treatments, and found that EMT occurred after treatment with oxaliplatin [27] and that the downregulation of CSCs and inhibition of stemness occur in the sensitization of HCC to oxaliplatin [18]. We then constructed oxaliplatin-resistant HCC models and found increased expression of both CCN2 and Id-1 in the oxaliplatin-resistant HCC using cDNA microarrays [3]. In the present study, we showed that the expression of Id-1 and CCN2 were closely correlated with malignancy in HCC patients, especially in the patients resistant to TACE. The adverse role of Id-1 and CCN2, and the mutual regulatory role were also explored in HCC patients and validated in HCC with primary and acquired oxaliplatin resistance.

Id-1 plays an important role in a number of cellular processes, including cellular development, senescence, differentiation, angiogenesis, and migration [28, 29]. The ability of Id-1 to drive self-renewal was first established in neural stem cells [30]. However, Id-1 is also significantly associated with breast [31], pancreas [32], cervical [33], ovarian [34], and colorectal cancers in humans [35]. In HCC, Id-1 expression is related to HCC dedifferentiation [36], and might serve as a potential prognostic marker for HBV-related HCC [37]. Recently, Sharma et al. [12] found that Id-1 promoted metabolic reprogramming in HCC cells. In the current study, we found that Id-1 was closely related to oxaliplatin resistance in mouse models of HCC. Increased Id-1 expression was likewise demonstrated in HCC and oxaliplatin-resistant HCC, and was significantly correlated with tumor malignancy and poor prognosis.

Members of the CCN family of secreted proteins typically interact with various cytokines to elicit cell proliferation, adhesion, invasion, migration, embryonic development, angiogenesis, wound healing, fibrosis, or inflammation. In HCC, we proved that CCN2 expression is an independent factor associated with shorter OS and prolonged CCR, and upregulation of CCN2 significantly enhanced oxaliplatin resistance. Hou et al. [38] showed that CCN2 is related to the formation of bone metastases in HCC, whereas other studies have indicated that CCN2 could serve as a potential prognostic biomarker [39] and that the inhibition of CCN2 blocks the progression of HCC [40]. Previously, we found a positive relationship between Id-1 and CCN2 expression in HCC by cDNA microarray [3], which was confirmed in the present study. Exploration of the forward regulatory mechanism of CCN2 and Id-1 using cDNA microarrays revealed that Id-1 and the MAPK signaling pathway were downstream of CCN2 signaling. Hence, Id-1 could partially upregulate CCN2 in a positive feedback manner. We likewise demonstrated that MAPK/Erk/Id-1 signaling was one of the most important autocrine signaling pathways regulated by CCN2 in oxaliplatin-resistant models and that the mechanism is involved in stemness maintenance and oxaliplatin resistance in HCC.

The MAPK/Erk signaling pathway plays a central role in HCC progression and is crucial for HCC proliferation [41]. Many studies have implicated the MAPK/Erk pathway in both the development and progression of HCC and indicated that activation of the MAPK/Erk pathway correlates with a poor prognosis in human HCC [42]. In the present study, we demonstrated that the activation of MAPK/Erk signaling and the upregulation of Id-1 were related to oxaliplatin resistance and enhanced stemness in HCC and that oxaliplatin treatment significantly activated MAPK/Erk signaling and upregulated Id-1 expression in a time- and dose-dependent manner. Further, when we silenced endogenous CCN2 expression or treated HCC cell lines with a combination of sorafenib or U0126 and oxaliplatin, MAPK/Erk signaling was impaired and Id-1 expression was downregulated. Hence, we believe that Id-1 could be an effector molecule of CCN2 and that the upregulation of CCN2 and Id-1 could serve as molecular markers of chemo-resistance. Additionally, the use of additive or synergistic therapies in the subgroup of HCC with highly activated CCN2/MAPK/Erk/Id-1 signaling could enhance current knowledge of chemotherapy and aid in developing individual treatments.

In summary, our study demonstrated that Id-1 and CCN2 were frequently upregulated in HCC patients and served as an independent prognostic factor associated with malignancy. The upregulation of Id-1 involved changes in the upregulation of CCN2 in HCC after oxaliplatin treatments and was accompanied by the activation of the MAPK/Erk signaling pathway. Thus, the combination of oxaliplatin with strategies designed to inhibit CCN2/MAPK/Erk signaling could provide a promising approach to ameliorating HCC progression, especially oxaliplatin resistance.

## Conclusions

From our experimental results and our review of the literature, we propose the following conclusions: (1) Id-1 and CCN2 in HCC are associated with a malignant phenotype and poor prognosis in HCC; (2) enhanced stemness is profoundly influenced by the expression levels of Id-1 and CCN2; (3) CCN2 significantly activates the MAPK/Erk/Id-1 signaling pathway and Id-1 positive feedback amplified the expression of CCN2; (4) oxaliplatin combined with CCN2/MAPK/Erk signaling inhibition results in improved treatment effects in HCC.

## Electronic supplementary material

Below is the link to the electronic supplementary material.

Supplementary material 1 (TIFF 4930 kb)

Supplementary material 2 (TIFF 7357 kb)

Supplementary material 3 (JPEG 1241 kb)

Supplementary material 4 (TIFF 799 kb)

Supplementary material 5 (TIFF 9459 kb)

Supplementary material 6 (TIFF 1668 kb)

Supplementary material 7 (JPEG 1061 kb)

Supplementary material 8 (TIFF 5199 kb)

Supplementary material 9 (JPEG 570 kb)

Supplementary material 10 (TIFF 2062 kb)

Supplementary material 11 (TIFF 4780 kb)

Supplementary material 12 (TIFF 7658 kb)

Supplementary material 13 (TIFF 6564 kb)

Supplementary material 14 (TIFF 4270 kb)

Supplementary material 15 (TIFF 29022 kb)

Supplementary material 16 (TIFF 9108 kb)

Supplementary material 17 (DOC 66 kb)

Supplementary material 18 (DOC 116 kb)

## Abbreviations

HCC: Hepatocellular carcinoma
CTGF: Connective tissue growth factor
Id-1: Inhibitor of DNA binding protein 1
HLH: Helix–loop–helix
GEO: Gene Expression Omnibus
ALDH: Aldehyde dehydrogenase
EpCAM: Epithelial cell adhesion molecule
EMT: Epithelial mesenchymal transition
TACE: Transcatheter arterial chemoembolization
OS: Overall survival
CCR: Cumulative recurrence rates
HBV: Hepatitis B virus
HBsAg: HBV surface antigen
AFP: Alpha-fetoprotein
ALT: Alanine aminotransferase
OPN: Osteopontin
GO: Gene Ontology
KEGG: Kyoto Encyclopedia of Genes and Genomes
CSCs: Cancer stem cells
