# Supplementary figures and images for: CCN2–MAPK–Id-1 loop feedback amplification is involved in maintaining stemness in oxaliplatin-resistant hepatocellular carcinoma
Source: Hepatol Int. 2019 Jun 27;13(4):440–53. doi: 10.1007/s12072-019-09960-5 (PMC6661033; doi:10.1007/s12072-019-09960-5)

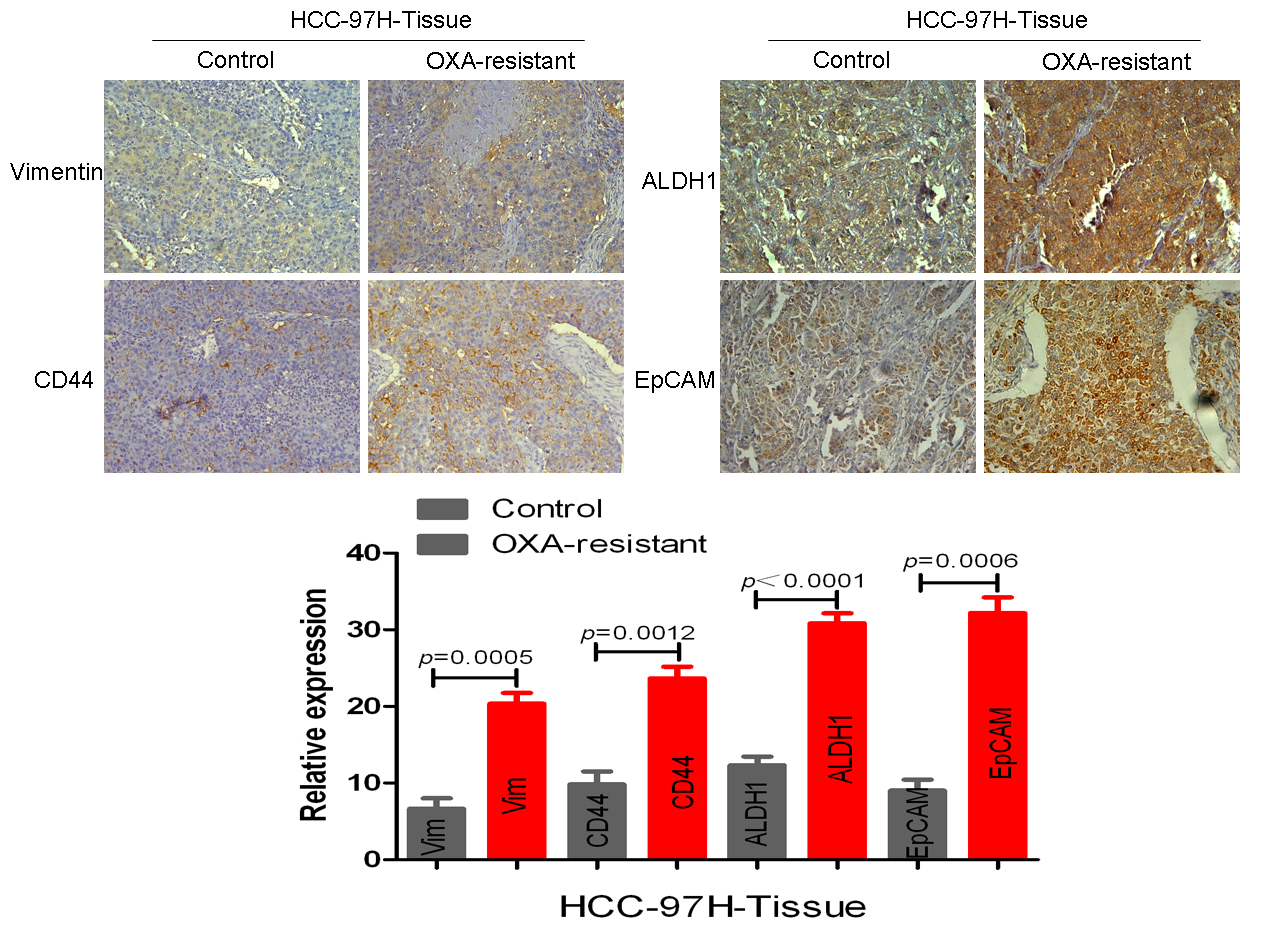

Supplement: Supplementary file 1 — Supplementary material 1 (TIFF 4930 kb) [file 12072_2019_9960_MOESM1_ESM.tif]

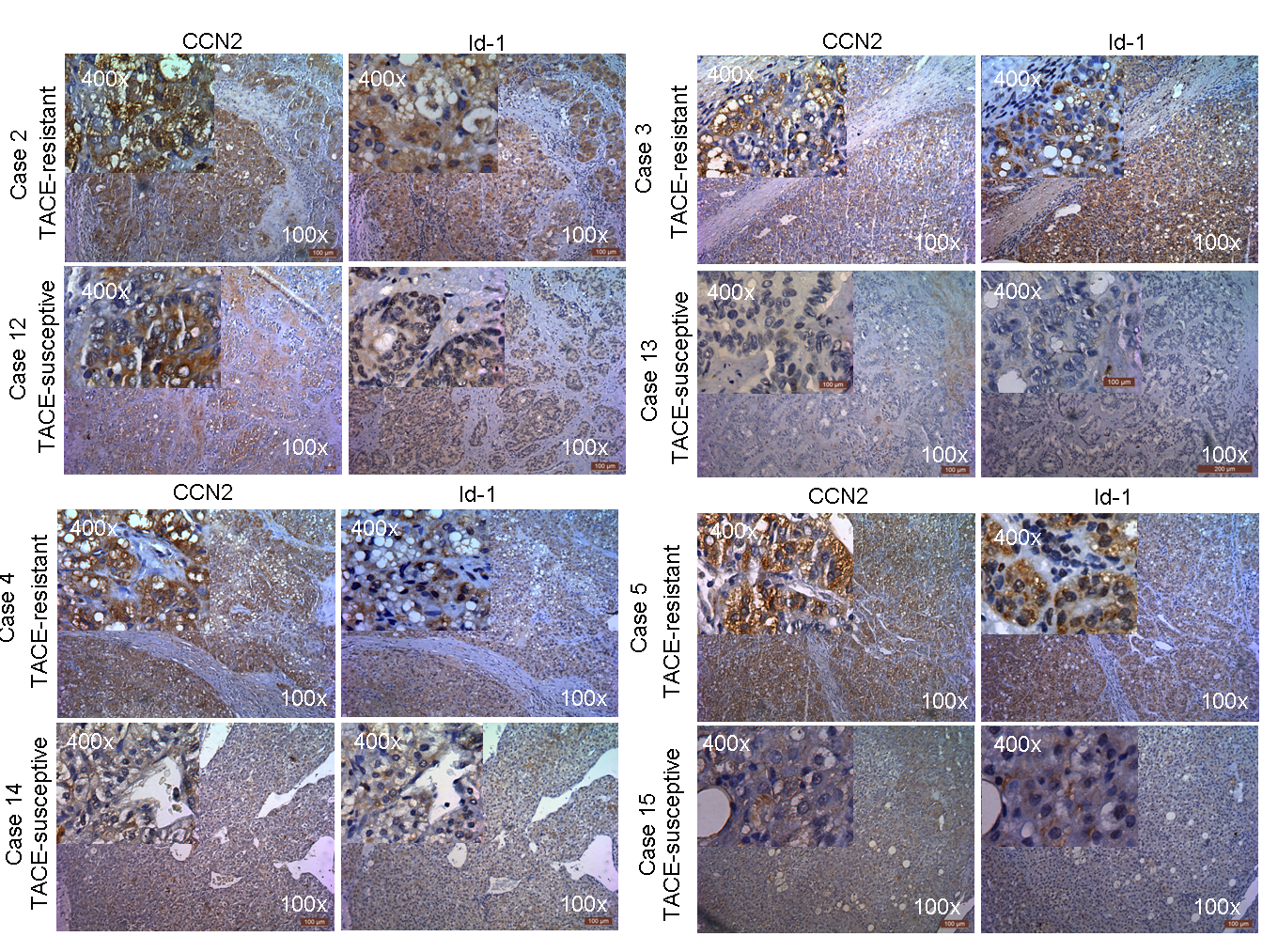

Supplement: Supplementary file 2 — Supplementary material 2 (TIFF 7357 kb) [file 12072_2019_9960_MOESM2_ESM.tif]

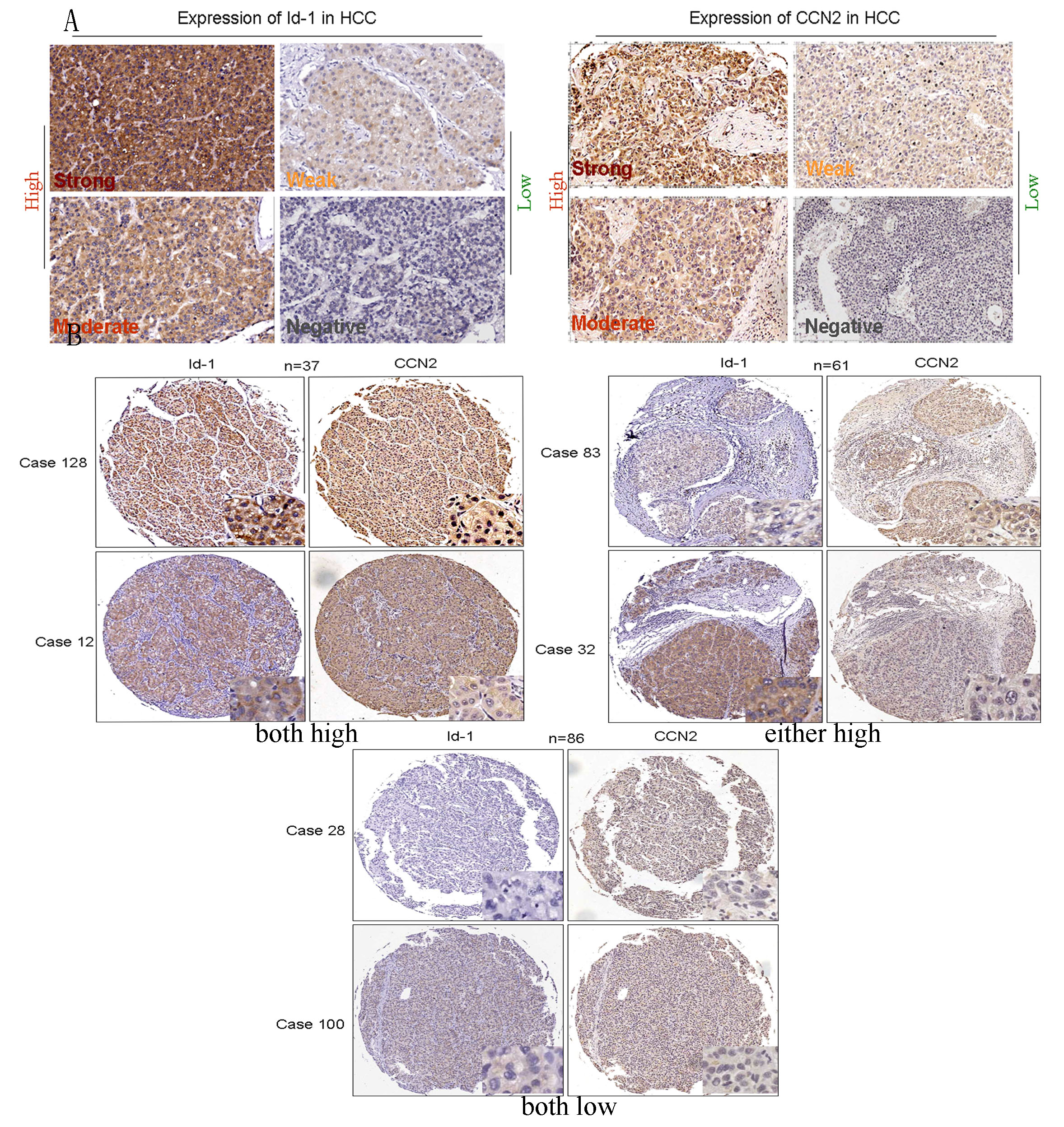

Supplement: Supplementary file 3 — Supplementary material 3 (JPEG 1241 kb) [file 12072_2019_9960_MOESM3_ESM.jpg]

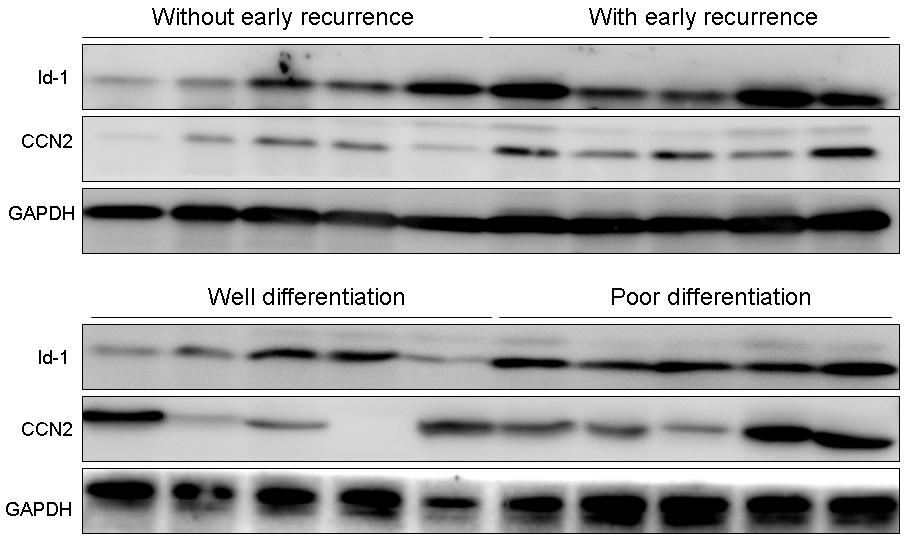

Supplement: Supplementary file 4 — Supplementary material 4 (TIFF 799 kb) [file 12072_2019_9960_MOESM4_ESM.tif]

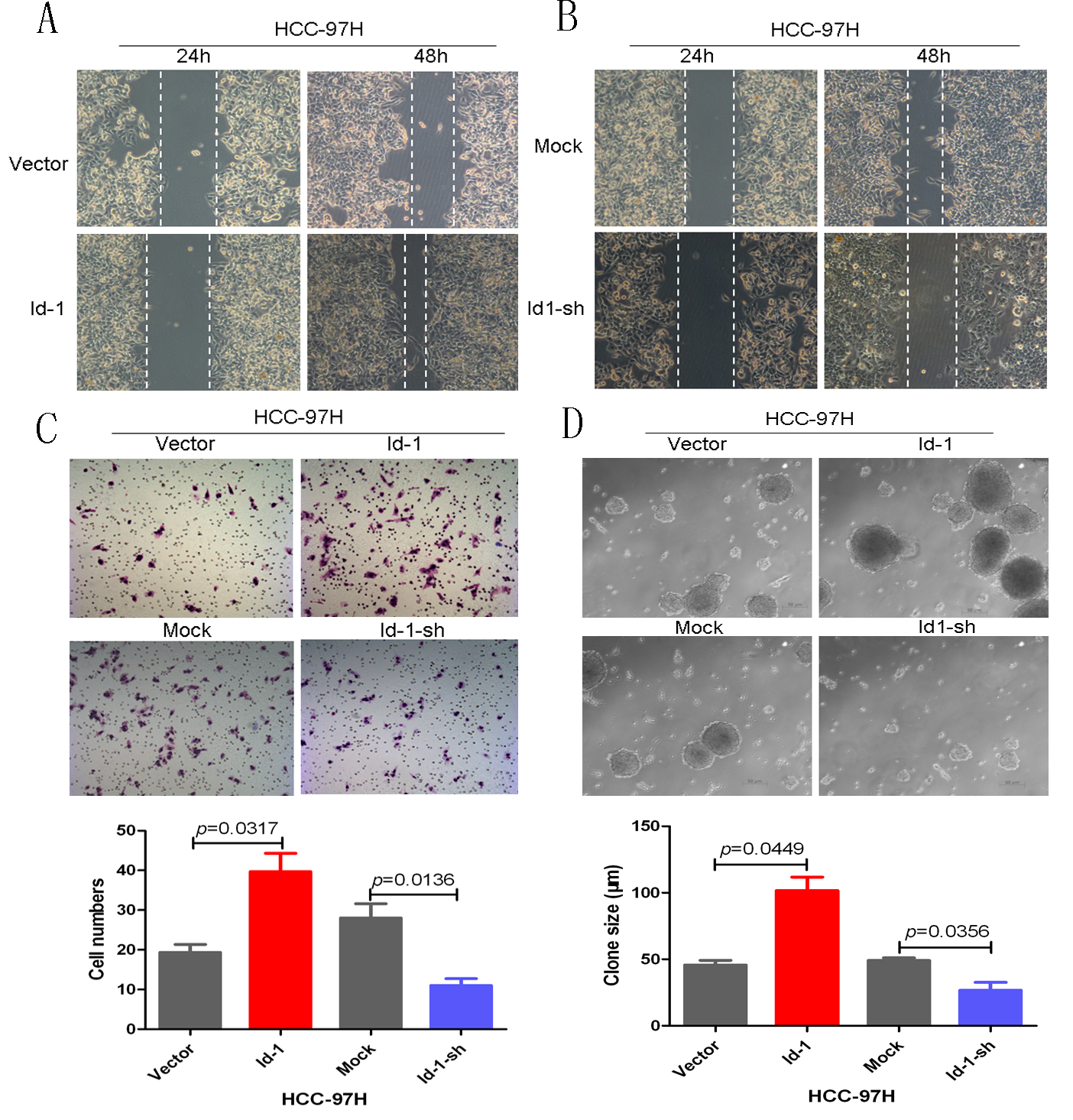

Supplement: Supplementary file 5 — Supplementary material 5 (TIFF 9459 kb) [file 12072_2019_9960_MOESM5_ESM.tif]

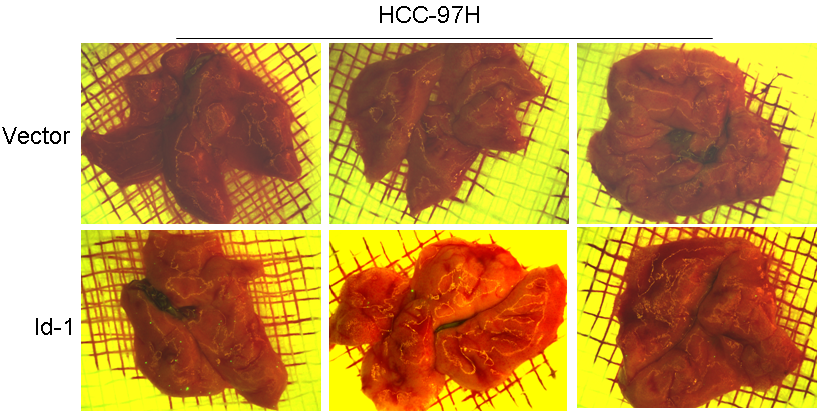

Supplement: Supplementary file 6 — Supplementary material 6 (TIFF 1668 kb) [file 12072_2019_9960_MOESM6_ESM.tif]

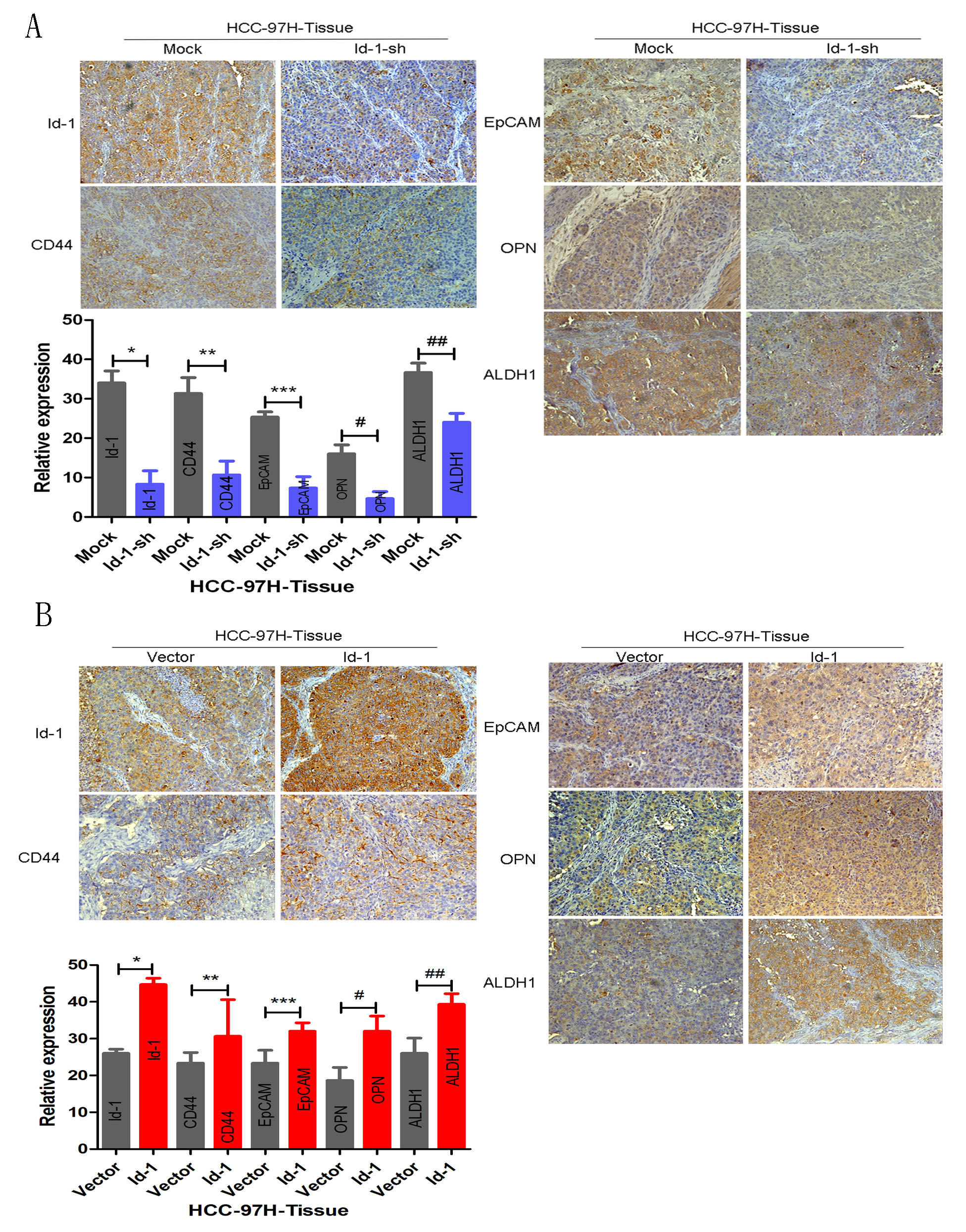

Supplement: Supplementary file 7 — Supplementary material 7 (JPEG 1061 kb) [file 12072_2019_9960_MOESM7_ESM.jpg]

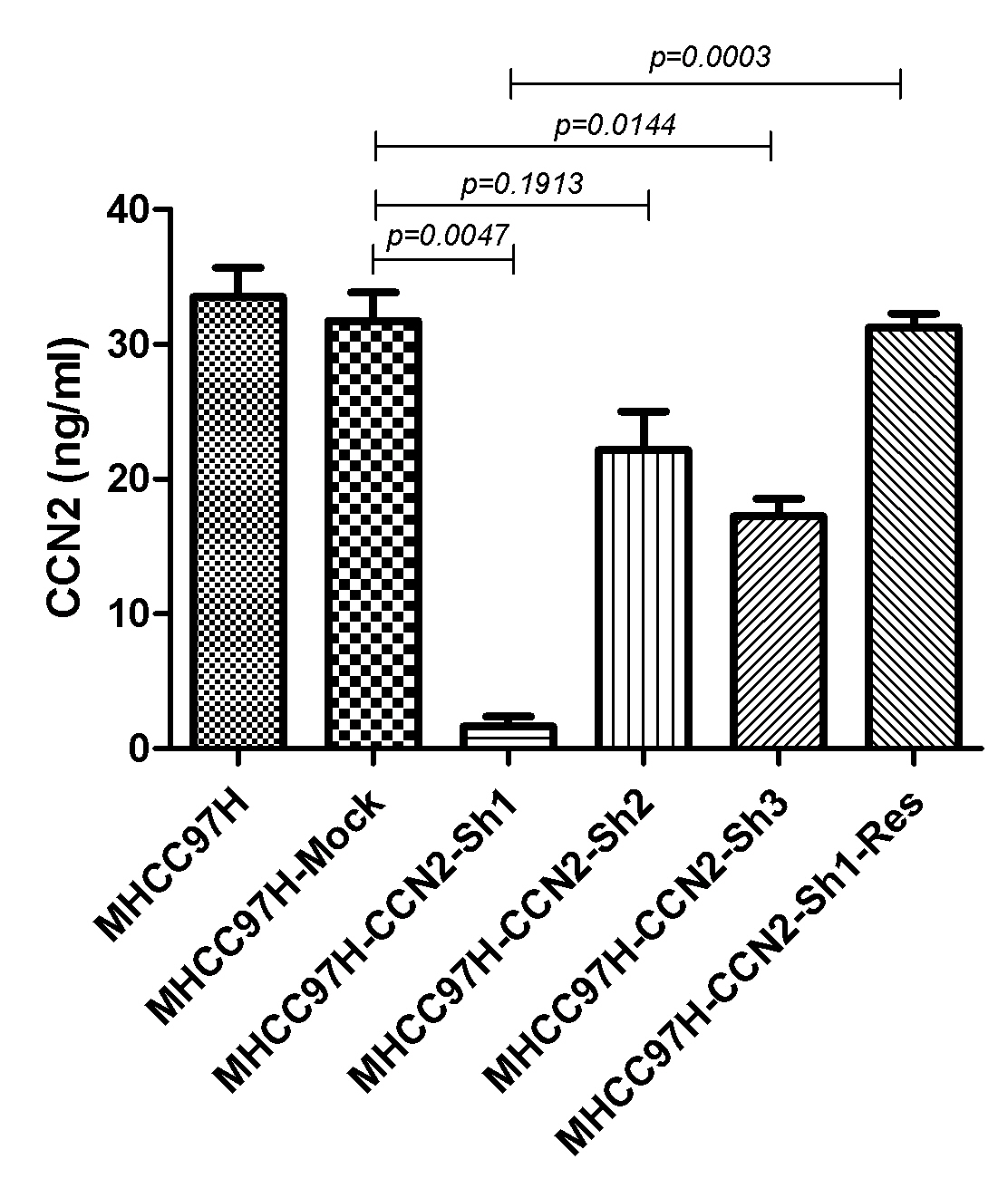

Supplement: Supplementary file 8 — Supplementary material 8 (TIFF 5199 kb) [file 12072_2019_9960_MOESM8_ESM.tif]

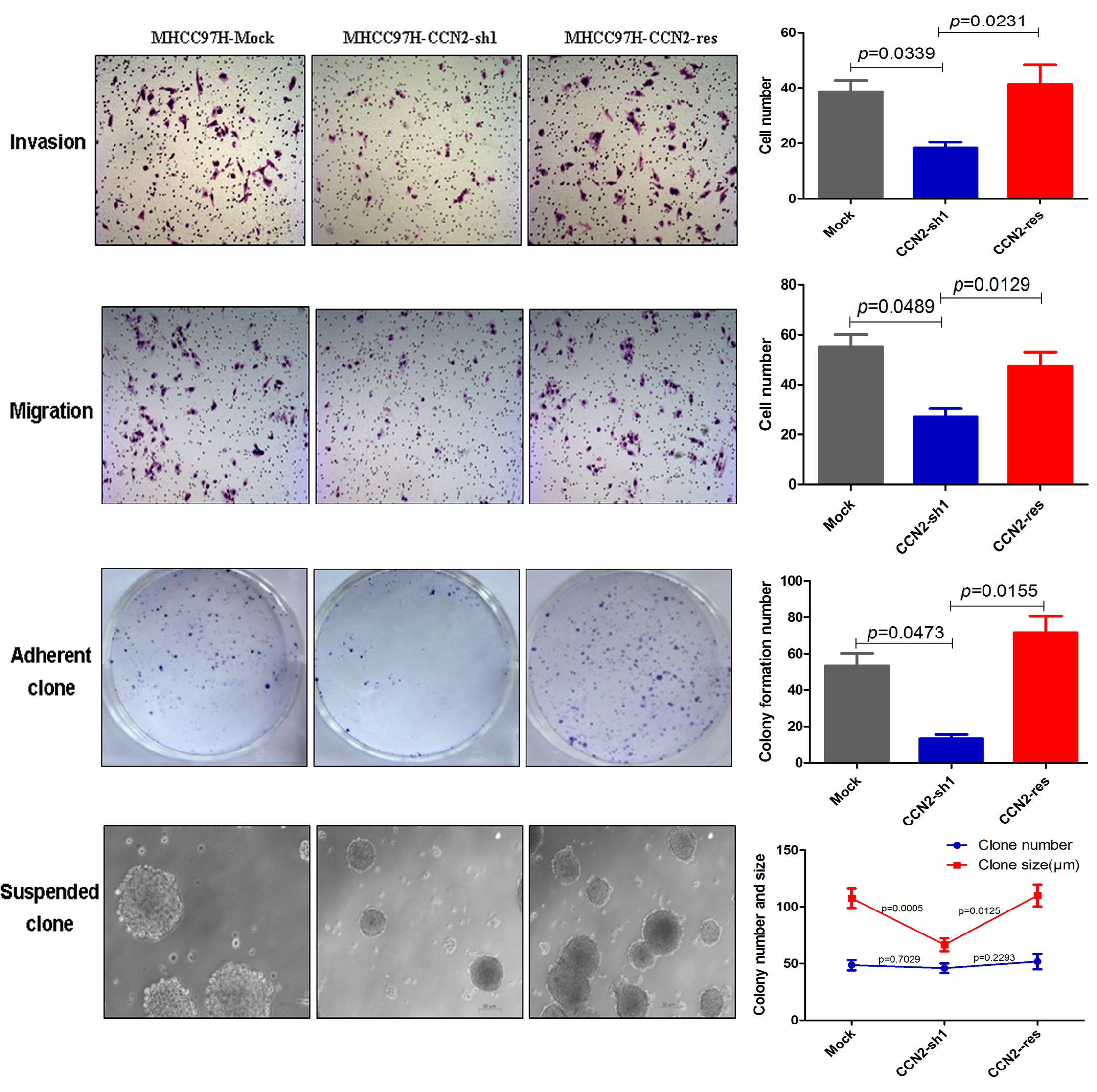

Supplement: Supplementary file 9 — Supplementary material 9 (JPEG 570 kb) [file 12072_2019_9960_MOESM9_ESM.jpg]

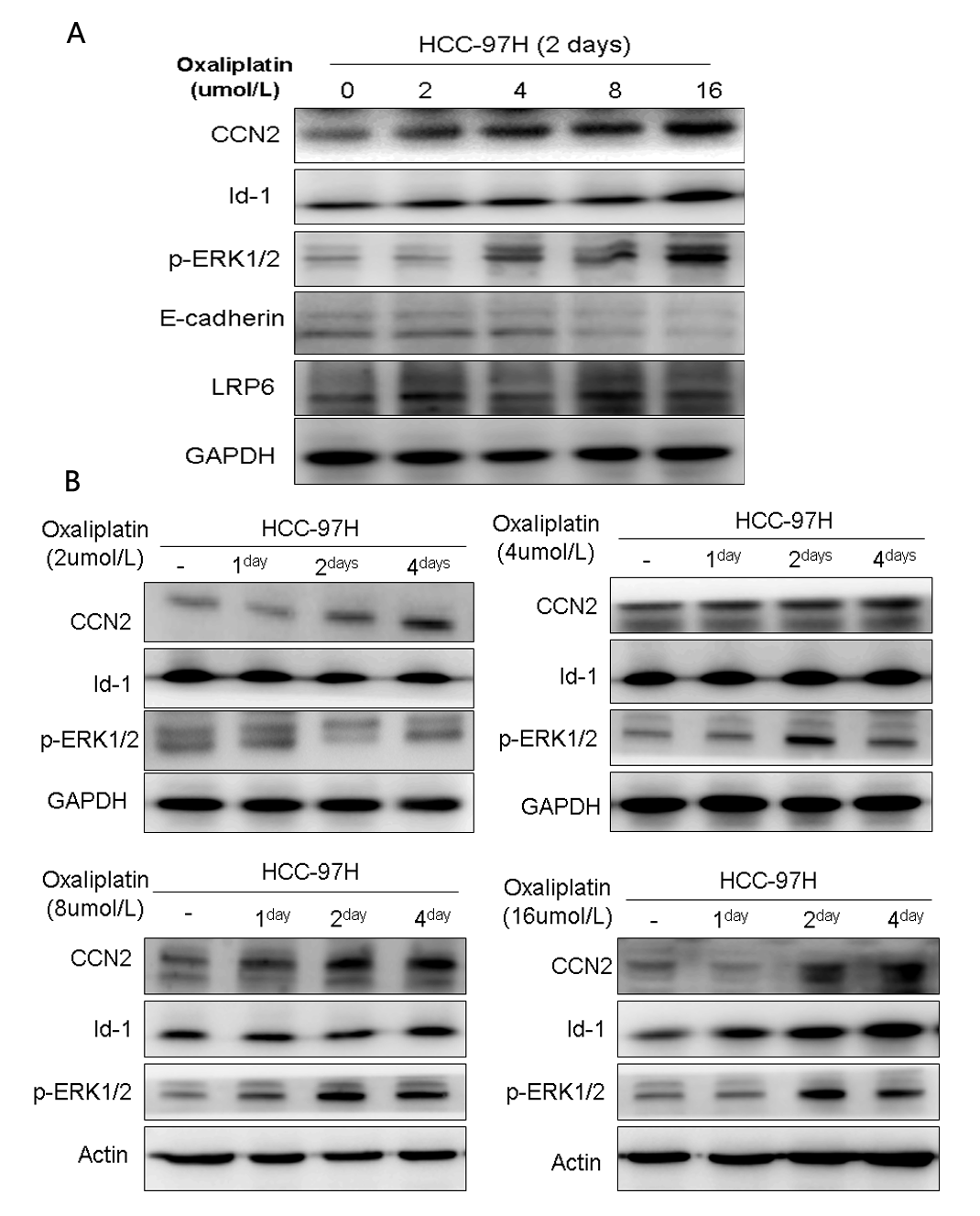

Supplement: Supplementary file 10 — Supplementary material 10 (TIFF 2062 kb) [file 12072_2019_9960_MOESM10_ESM.tif]

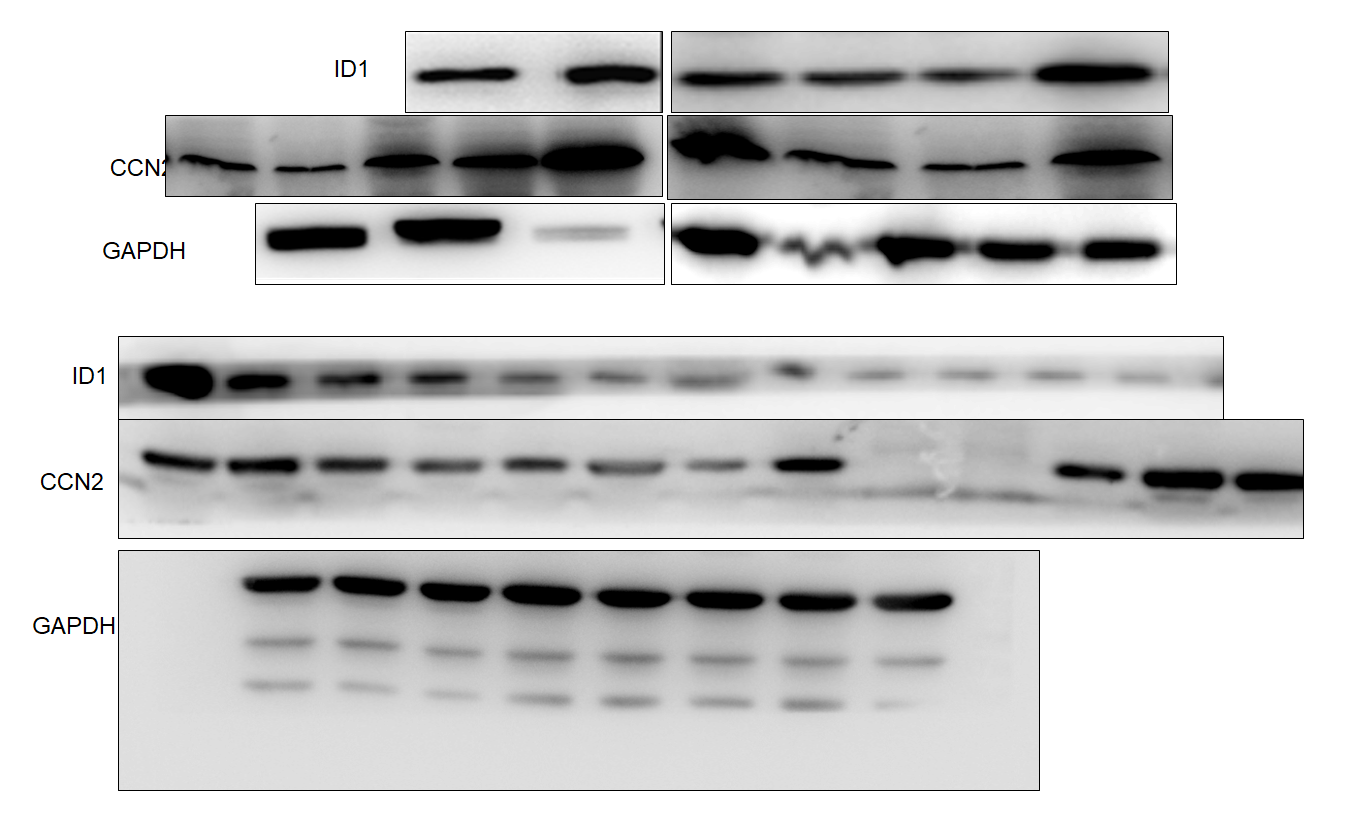

Supplement: Supplementary file 11 — Supplementary material 11 (TIFF 4780 kb) [file 12072_2019_9960_MOESM11_ESM.tif]

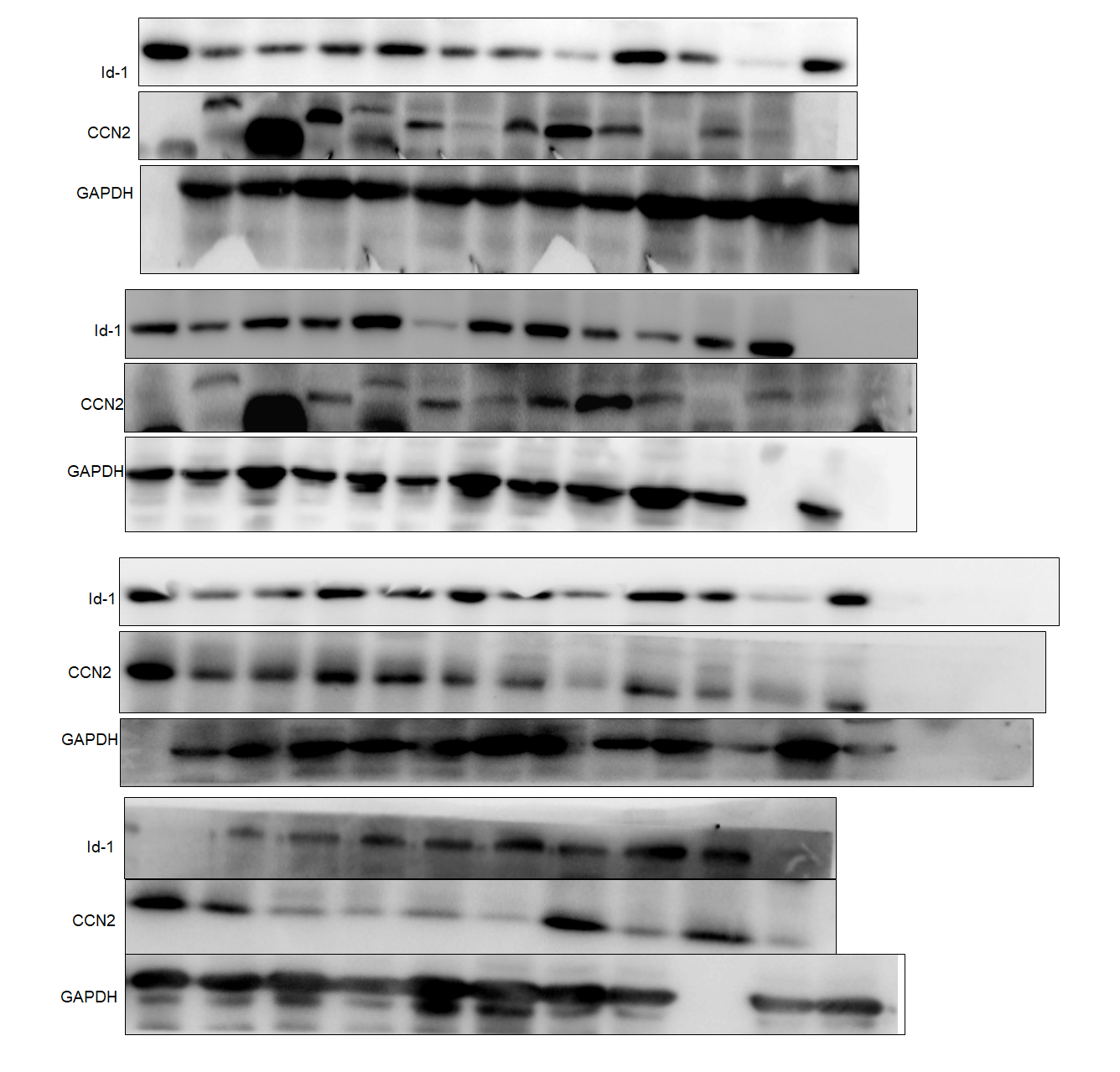

Supplement: Supplementary file 12 — Supplementary material 12 (TIFF 7658 kb) [file 12072_2019_9960_MOESM12_ESM.tif]

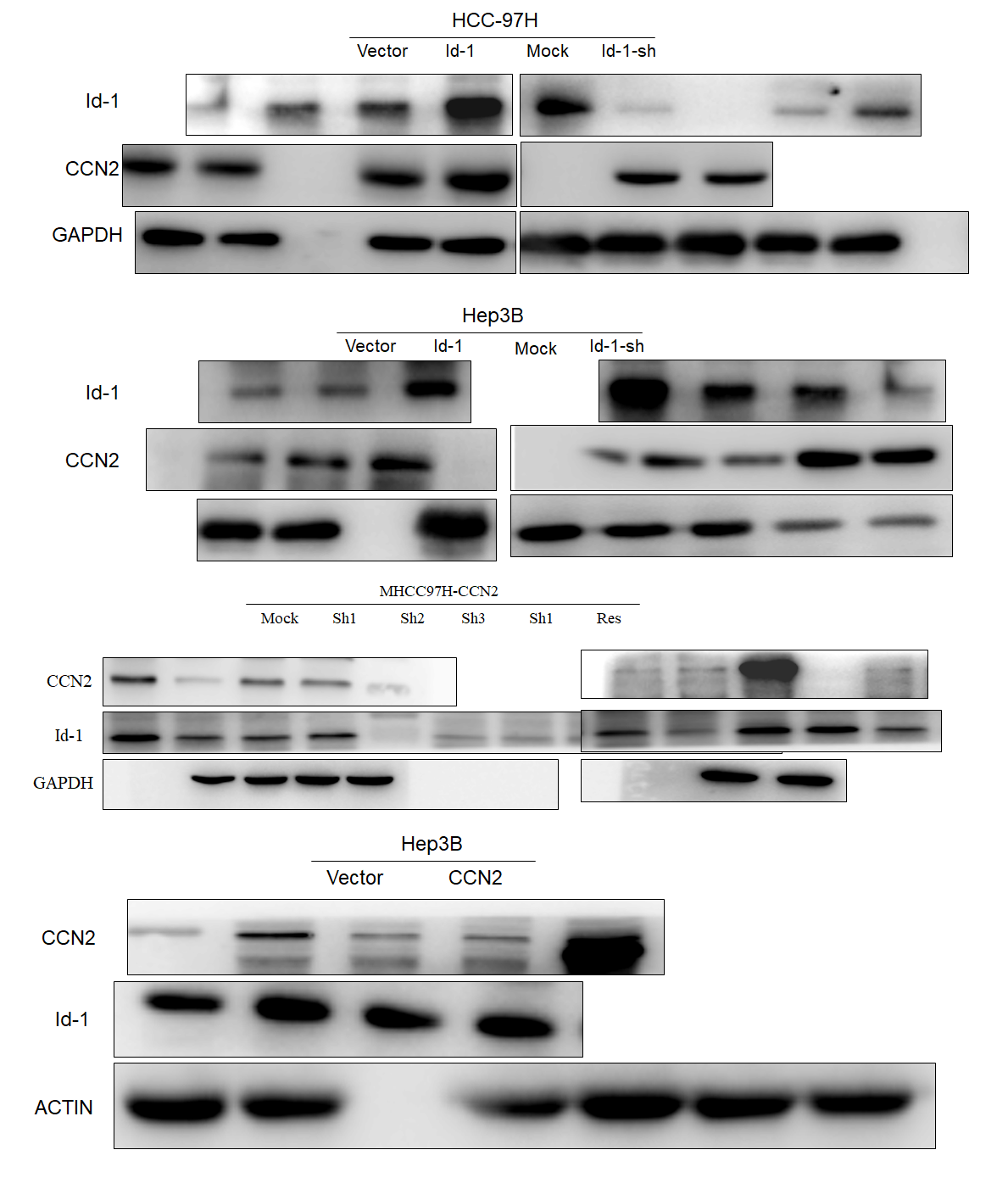

Supplement: Supplementary file 13 — Supplementary material 13 (TIFF 6564 kb) [file 12072_2019_9960_MOESM13_ESM.tif]

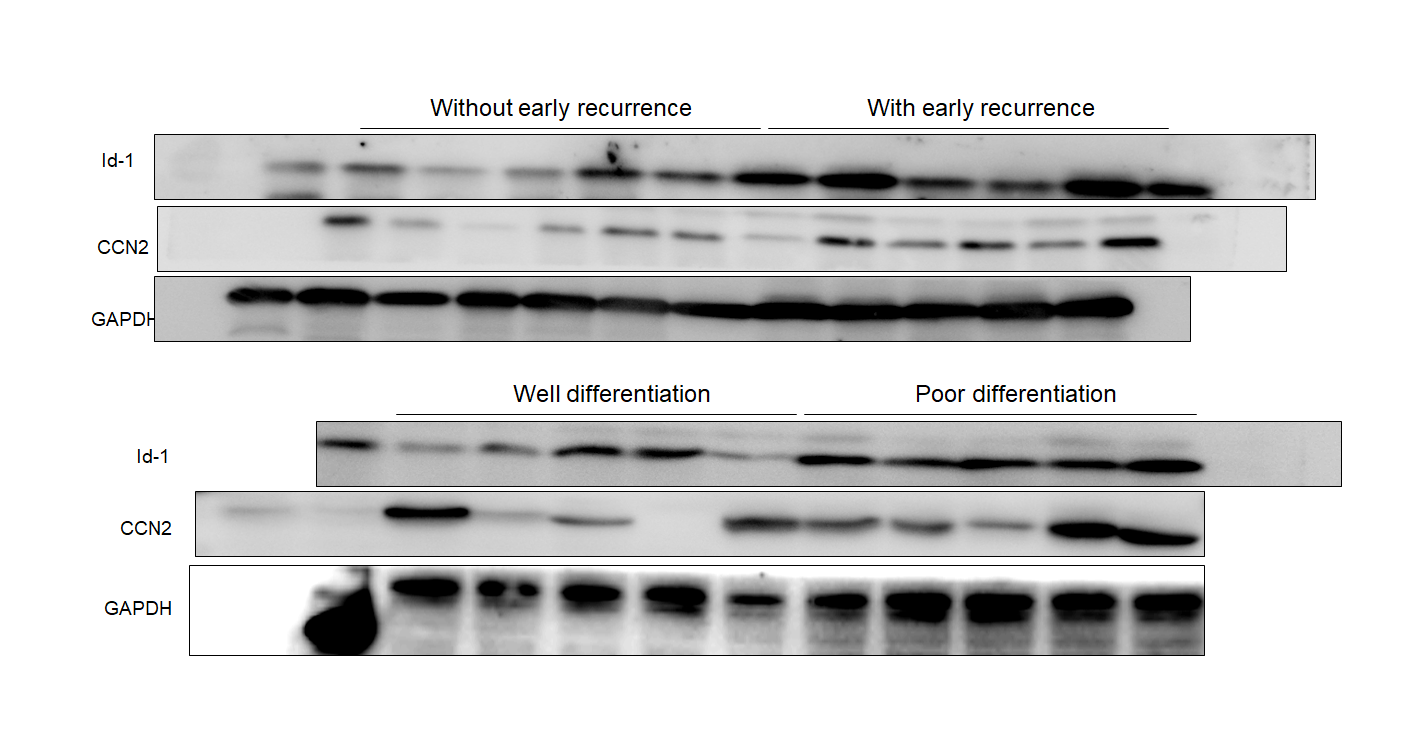

Supplement: Supplementary file 14 — Supplementary material 14 (TIFF 4270 kb) [file 12072_2019_9960_MOESM14_ESM.tif]

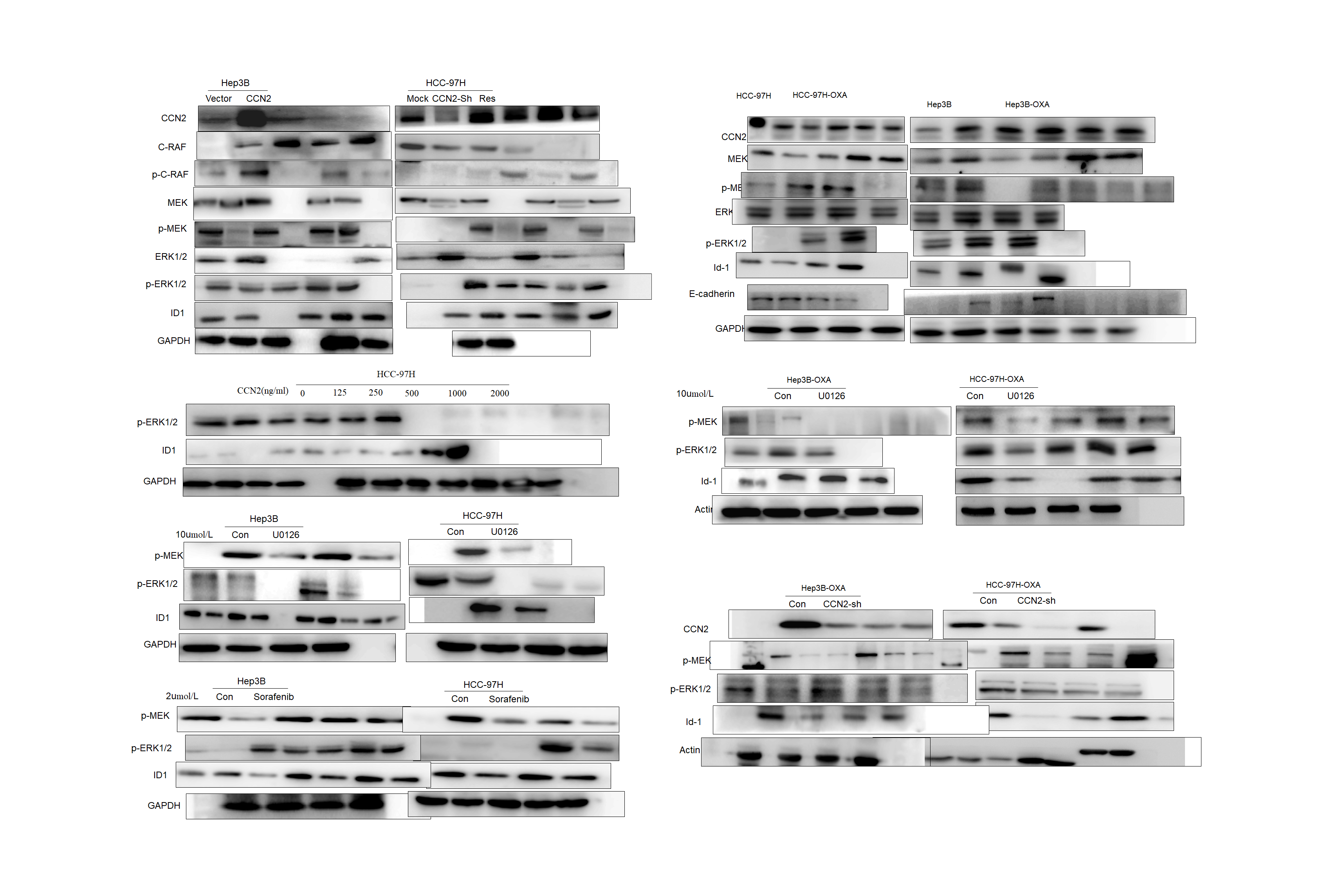

Supplement: Supplementary file 15 — Supplementary material 15 (TIFF 29022 kb) [file 12072_2019_9960_MOESM15_ESM.tif]

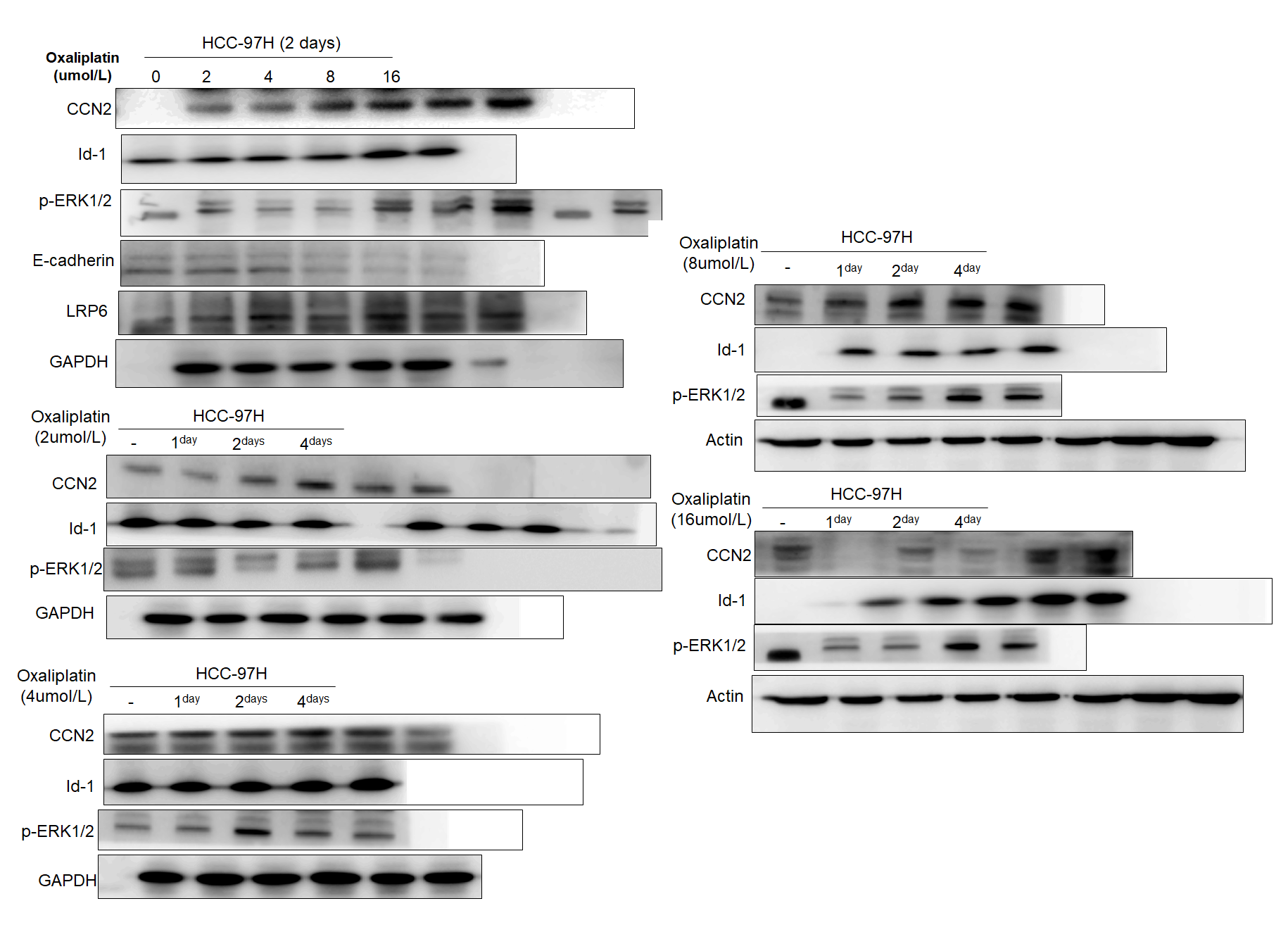

Supplement: Supplementary file 16 — Supplementary material 16 (TIFF 9108 kb) [file 12072_2019_9960_MOESM16_ESM.tif]
