# Supplementary material for: CCN2–MAPK–Id-1 loop feedback amplification is involved in maintaining stemness in oxaliplatin-resistant hepatocellular carcinoma
Source: Hepatol Int. 2019 Jun 27;13(4):440–53. doi: 10.1007/s12072-019-09960-5 (PMC6661033; doi:10.1007/s12072-019-09960-5)
Supplement: Supplementary file 17 — Supplementary material 17 (DOC 66 kb) [file 12072_2019_9960_MOESM17_ESM.doc]

**Supplementary Materials and Methods**

**Cell Lines and Animals**

The HCC-LM3 and HCC-97H human HCC cell lines with high metastatic potential were established at the Fudan University. The LO_2_ human liver cell line and the PLC, HepG_2_, Huh7, and Hep3B human HCC cell lines with low metastatic potential were obtained from the Chinese Academy of Science. All cells were maintained in Dulbecco's Modified Eagle Medium (DMEM) (GICBO, Grand Island, NY) supplemented with 10% fetal bovine serum (GICBO) at 37°C in a humidified incubator with 5% CO_2_.

Male BALB/c nu/nu mice and C57 mice (aged 4–6 weeks and weighing approximately 20 g) were maintained under standard pathogen-free conditions (SLRC, Shanghai, China). The experimental protocol was approved by the Shanghai Medical Experimental Animal Care Commission.

**Reagents and Antibodies**

Oxaliplatin, sorafenib, and the MEK1/2 inhibitor U1026 were obtained from Selleckchem (Houston, USA). Recombinant human CTGF was obtained from Peprotech (New Jersey, USA). The Cell Counting Kit-8 was obtained from Dojindo (Kumamoto, Japan). The primary antibodies used in this study and their respective concentrations are listed in Supplementary Table 4.

**Dose- and Time-Response of oxaliplatin in HCC Cells**

HCC-97H cells were allowed to adhere and were incubated with 1, 2, 4, 8, and 16 μmol/L oxaliplatin for 2 days in T25 cell culture flasks. Afterward, 2 μmol/L oxaliplatin was used to separately treat the HCC-97H cells for 1, 2, and 4 days. Proteins were extracted in modified radioimmunoprecipitation assay (RIPA) buffer supplemented with protease inhibitor cocktail (Sigma-Aldrich, St. Louis, MO, USA).

**Construction of Oxaliplatin-resistant HCC cell lines**

HCC-97H and Hep3B cells were grown to 80% confluence, harvested with trypsin, and plated in T25 cell culture flasks (5 × 10^5^ cells per flask). After 24 h, the medium was replaced with DMEM containing 10% FBS and either 1 μmol/L or 0.5 μmol/L oxaliplatin. After 48 h, the medium was changed and drug treatment was terminated. Cells were allowed to recover, and when the surviving populations reached 80% confluence, the cells were passaged and exposed to a greater concentration of oxaliplatin (2 μmol/l or 1 μmol/l) again for 48 h. The above procedure was repeated using increasing concentrations of oxaliplatin. Once 10 μmol/l oxaliplatin was added to the HCC-97H cells and 5 μmol/l oxaliplatin was added to the Hep3B cells and the HCC cells were stably resistant to oxaliplatin, the cells were re-named MHCC97H-OXA and Hep3B-OXA, respectively.

**RNA Extraction and qRT-PCR**

Total RNA was extracted from HCC cells using the TRIzol® reagent (Invitrogen, Carlsbad, CA, USA). The primers used for the amplification of human genes are provided in Supplementary Table 5.

**Cell Proliferation Assay**

Cells were cultured in 96-well plates (4 × 10^3^ cells/well) and exposed to increasing concentrations of oxaliplatin (0, 0.5, 1, 2, 4, 8, 16, 32, 64, and 128 μmol/L) for 72 h. The constant concentration of sorafenib (2 µmol/L) or the MEK1/2 inhibitor U0126 (5 µmol/L) was combined. The relative number of cells was calculated using the Cell Counting Kit-8 (Dojindo). The results were expressed as the absorbance of each well at 450 nm (OD 450).

**Cell Migration, Matrigel® Invasion, and Sphere** **Formation Ability Assays**

Cell migration, invasion, and colony formation assays were performed as previously described .

**Construction of Tissue Microarrays, Immunohistochemistry, and Immunoblotting**

Tissue microarrays were constructed by Shanghai Biochip Co, Ltd. as previously described . Paraffin-embedded tissue sections (4 μm) were prepared according to established methods. Immunohistochemistry and western blotting were performed as previously described . The concentration of extracted protein was determined using a BCA Protein Assay Kit (Beyotime. Shanghai, China).

**Reference**

1. Bu Y, Jia QA, Ren ZG, Xue TC, Zhang QB, Zhang KZ, et al. The herbal compound Songyou Yin (SYY) inhibits hepatocellular carcinoma growth and improves survival in models of chronic fibrosis via paracrine inhibition of activated hepatic stellate cells. Oncotarget. 2015. Epub 2015/10/31.

2. Wan S, Zhao E, Kryczek I, Vatan L, Sadovskaya A, Ludema G, et al. Tumor-associated macrophages produce interleukin 6 and signal via STAT3 to promote expansion of human hepatocellular carcinoma stem cells. Gastroenterology. 2014;147(6):1393-404. Epub 2014/09/03.

3. Ke AW, Shi GM, Zhou J, Wu FZ, Ding ZB, Hu MY, et al. Role of overexpression of CD151 and/or c-Met in predicting prognosis of hepatocellular carcinoma. Hepatology. 2009;49(2):491-503. Epub 2008/12/10.

4. Jia QA, Ren ZG, Bu Y, Wang ZM, Zhang QB, Liang L, et al. Herbal Compound "Songyou Yin" Renders Hepatocellular Carcinoma Sensitive to Oxaliplatin through Inhibition of Stemness. Evid Based Complement Alternat Med. 2012;2012:908601. Epub 2013/01/18.

**Supplementary Table 1.** Correlations between Id-1 and clinicopathologic characteristics of patients with HCC

**Supplementary Table 2.** Correlations between CCN2 and clinicopathologic characteristics of patients with HCC

**Supplementary Table 3.** Univariate analysis of factors associated with survival and recurrence in patients with HCC

**Supplementary Table 4.** Multivariate analysis of factors associated with survival and recurrence in patients with HCC

**Supplementary Table 5.** Primary antibodies used for western blotting and immunohistochemistry.

**Supplementary Table 6.** Sequences of primers used for qRT-PCR.

**Supplementary Table 7.** shRNA target sequences and the downregulation efficiency of Id-1

**Supplementary Fig. 1** Significantly increased expression of vimentin, CD44, ALDH1, and EpCAM were upregulated in subcutaneous oxaliplatin-resistant tumors.

**Supplementary Fig. 2** Increased expression of Id-1 and CCN2 was shown in TACE-resistant patients compared to TACE-susceptible patients.

**Supplementary Fig. 3** Patients (184) were sorted according to the Id-1 and CCN2 expression levels. (*A*) Id-1 and CCN2 expression were divided four degree according to strong, moderate, weak, and negative expression. (*B*) Patients were classified into three subgroups according to CCN2 and Id-1 expression levels.

**Supplementary Fig. 4** Increased Id-1 and CCN2 expression in protein levels by immunoblotting found to be associated with poor differentiation and early recurrence of HCC

**Supplementary Fig. 5** The malignant characteristics of HCC-97H cells were also significantly enhanced after overexpression of Id-1 but inhibited after Id-1 silencing. (*A, B*) Increased Id-1 expression significantly enhanced migration ability in MHCC-97H-Id-1 cells, whereas migration ability was reduced after the silencing of Id-1. (*C*) Increased Id-1 expression significantly enhanced the invasion ability, whereas the invasion ability was reduced after the interference of Id-1. (*D*) The sphere formation ability was also enhanced after overexpression of Id-1 but the ability was reduced after the interference of Id-1.

**Supplementary Fig. 6** HCC-97H cells overexpressing Id-1 exhibited pulmonary metastasis, whereas the vector group was negative for lung metastases.

**Supplementary Fig. 7** The expression of Id-1 was positive related to the CCN2 expression. (*A*) Immunohistochemical staining revealed that the silencing of endogenous Id-1 significantly decreased the expression of CD44, EpCAM, OPN, and ALDH1 in HCC tissues from mice with subcutaneous tumors, (*B*) while, the stemness associated markers were significantly up-regulated after Id-1 expression was over-expressed compared with Vector group.

**Supplementary Fig. 8** The silenced efficiency of CCN2 was evaluated compared with MHCC-97H-mock cells, and restoration of CCN2 expression could rescue the altered expression of CCN2.

**Supplementary Fig. 9** The downregulation of CCN2 expression significantly impaired the invasiveness, migration, proliferation and sphere formation abilities compared with the controls.

**Supplementary Fig. 10** The MAPK/Erk signaling pathway was activated after oxaliplatin treatment in a dose- and time- dependent manner. (*A*) HCC-97H cells treated with oxaliplatin exhibited variations in CCN2, Id-1, LRP6, p-Erk, and E-cadherin in a dose-dependent manner, with the most significant changes occurring at the highest concentration. (*B*) HCC-97H cells treated with oxaliplatin over a time gradient also exhibited the associated variation in CCN2, Id-1, and p-Erk, with the most significant changes occurring at the maximal time.
