# Supplementary material for: CCN2–MAPK–Id-1 loop feedback amplification is involved in maintaining stemness in oxaliplatin-resistant hepatocellular carcinoma
Source: Hepatol Int. 2019 Jun 27;13(4):440–53. doi: 10.1007/s12072-019-09960-5 (PMC6661033; doi:10.1007/s12072-019-09960-5)
Supplement: Supplementary file 18 — Supplementary material 18 (DOC 116 kb) [file 12072_2019_9960_MOESM18_ESM.doc]

Supplementary Tab. 1

Correlations between Id-1 and clinicopathology feature in 184 patients with HCC

| Variable | No. of Patient | | | Variable | No. of Patient | | |
| --- | --- | --- | --- | --- | --- | --- | --- |
|  | Id-1^high^ | Id-1^low^ |  |  | Id-1^high^ | Id-1^low^ |  |
| Age, y  ≥53  ＜53 | 33  33 | 55  63 | 0.659 | Tumor dimension  ≥5cm  ＜5cm | 15  51 | 30  88 | 0.683 |
| Sex  Men  Women | 56  10 | 96  22 | 0.549 | No. of tumors  Multiple  single | 5  61 | 8  110 | 0.840 |
| HBsAg  Positive  Negative | 63  3 | 103  15 | 0.058 | Vascular invasion  Yes  No | 21  45 | 23  95 | 0.060 |
| Cirrhosis  Yes  No | 58  8 | 101  17 | 0.664 | Tumor encapsulation  Complete  None | 35  31 | 72  46 | 0.292 |
| Serum, AFP  ≥20  ＜20 | 37  29 | 71  47 | 0.587 | Tumor differentiation  Ⅰ-Ⅱ  Ⅲ-Ⅳ | 58  8 | 87  31 | **0.024** |
| Serum, ALT  ≥75  ＜75 | 8  58 | 11  107 | 0.550 |  |  |  |  |

AFP, alpha-fetoprotein; HBsAg, hepatitis B surface antigen；

Supplementary Tab. 2

Correlations between CCN2 and clinicopathology feature in 184 patients with HCC

| Variable | No. of Patient | | | Variable | No. of Patient | | |
| --- | --- | --- | --- | --- | --- | --- | --- |
|  | CCN2^high^ | CCN2^low^ |  |  | CCN2^high^ | CCN2^low^ |  |
| Age, y  ≥53  ＜53 | 32  39 | 56  57 | 0.553 | Tumor dimension  ≥5cm  ＜5cm | 20  51 | 25  88 | 0.353 |
| Sex  Men  Women | 61  10 | 91  22 | 0.348 | No. of tumors  Multiple  single | 10  61 | 3  110 | **0.004*** |
| HBsAg  Positive  Negative | 64  7 | 102  11 | 0.978 | Vascular invasion  Yes  No | 18  53 | 26  87 | 0.717 |
| Cirrhosis  Yes  No | 60  11 | 99  14 | 0.550 | Tumor encapsulation  Complete  None | 36  35 | 71  42 | 0.105 |
| Serum, AFP  ≥20  ＜20 | 42  29 | 66  47 | 0.920 | Tumor differentiation  Ⅰ-Ⅱ  Ⅲ-Ⅳ | 49  22 | 96  17 | **0.010** |
| Serum, ALT  ≥75  ＜75 | 11  60 | 11  102 | 0.241 |  |  |  |  |

AFP, alpha-fetoprotein; HBsAg, hepatitis B surface antigen；

*Fisher exact test

Supplementary Tab. 3

Univariate Analysis of Factors Associated With Survival and Recurrence (n=184)

| Variables | Overall Survival | | Cumulative Recurrence | |
| --- | --- | --- | --- | --- |
|  | Hazard ratio (95%  confidence interval)*a* | *P* value | Hazard ratio (95%  confidence interval)*a* | *P* value |
| Age, *y*, ≥53 vs ＜53 | 0.278(.780- 2.094) | 0.331 | 1.148(.735- 1.792) | 0.544 |
| Sex, male vs female | 0.917 (.480-1.720) | 0.788 | 1.216(.657-2.252) | 0.533 |
| HBsAg, positive VS negative | 0.834 (.380-1.831) | 0.650 | 1.060(.487-2.308) | 0.884 |
| Cirrhosis, no vs yes | 1.052(0.595-1.859) | 0.862 | 0.995(.589-1.681) | 0.986 |
| AFP, *ng/mL*, ≥20 vs ＜20 | 1.518 (0.906-2.543) | 0.113 | 1.255(0.795-1.980） | 0.330 |
| ALT | 0.954(0.435- 2.093) | 0.907 | 0.868(.417-1.805) | 0.740 |
| Tumor size | 2.565(1.526-4.312) | **0.000** | 2.686(1.671- 4.319) | **0.000** |
| No. of tumors | 5.925(2.882-12.177) | **0.000** | 5.194(2.645-10.201) | **0.000** |
| Vascular invasion, yes vs no | 2.458(1.465-4.123) | **0.001** | 1.801(1.099-2.952) | **0.020** |
| Tumor encapsulation | 1.520(0.931- 2.482) | 0.094 | 1.512(0.969-2.358) | 0.068 |
| Tumor differentiation | 1.526(0.866- 2.690) | 0.144 | 1.405(0.829-2.381) | 0.207 |
| Id-1, high vs low | 1.641(1.003-2.686) | **0.049** | 1.708(1.092-2.670) | **0.019** |
| CCN2, high vs low | 2.365(1.178-3.398) | **0.012** | 1.982(1.348-3.367) | **0.008** |

Supplementary Tab. 4

Multivarite Analysis of Factors Associated With Survival and Recurrence (n=184)

| Variables | Overall Survival | | Cumulative Recurrence | |
| --- | --- | --- | --- | --- |
|  | Hazard ratio (95%  confidence interval)*a* | *P* value | Hazard ratio (95%  confidence interval)*a* | *P* value |
| Tumor size | 2.367(1.371-4.085) | **0.002** | 2.625(1.588-4.341) | **0.000** |
| No. of tumors | 4.804(2.125-10.864) | **0.000** | 4.189(1.975-8.884) | **0.000** |
| Vascular invasion, yes vs no | 2.259(1.320-3.867) | **0.003** | 1.621(1.072-2.699) | **0.063** |
| Id-1, high vs low | 1.944(1.161-3.255) | **0.011** | 1.664(1.030-2.686) | **0.037** |
| CCN2, high vs low | 2.503(1.374- 3.790) | **0.008** | 2.500(1.551-4.031) | **0.000** |

Supplementary Tab. 5

Primary Antibodies for Western Blot, Immunohistochemistry

| Antibody | Concentration for WB | Concentration for IHC | Specifity | Company |
| --- | --- | --- | --- | --- |
| Id-1 | 1:1000 | 1:2000 | Rabbit monoclonal | Abcam |
| CCN2 | 1:800 | 1:500 | Rabbit monoclonal | Abcam |
| Vimentin | 1:1000 | 1:500 | Rabbit polyclonal | Abcam |
| ALDH1 | 1:1000 | 1:500 | Rabbit polyclonal | CST |
| CD44 | 1:600 | 1:500 | Rabbit monoclonal | Abcam |
| EpCAM | 1:400 | 1:500 | Rabbit monoclonal | CST |
| C-RAF | 1:2000 | - | Rabbit monoclonal | CST |
| p-C-RAF | 1:1000 | - | Rabbit monoclonal | Abcam |
| MEK | 1:1000 | - | Rabbit monoclonal | Abcam |
| p-MEK | 1:200 | - | Rabbit polyclonal | Epitomics |
| ERK1/2 | 1:1000 | - | Mouse monoclonal | Abcam |
| p-ERK1/2 | 1:1000 | - | Mouse monoclonal | Abcam |
| GAPDH | 1:1000 | - | Mouse monoclonal | Jackson |

Supplementary Tab. 6

Primers for qRT-PCR

| Gene | Forward primer (5’---3’) | Reverse primer (5’---3’) |
| --- | --- | --- |
| Id-1 | ACGACATGAACGGCTGTTACTC | CCGAGTTCAGCTCCAACTGAAG |
| CCN2 | GCATCTTCGGTGGTACGGTGTA | TGGACCAGGCAGTTGGCTCTA |
| Actin | CACCCAGCACAATGAAGATCAAGAT | CCAGTTTTTAAATCCTGAGTCAAGC |

Supplementary Tab. 7

vshRNA Target Sequences

|  | Target sequence |
| --- | --- |
| GFP-Id-1 |  |
| PLVT748 | CCGTATCTGCTTCGGGCTT |
| PLVT749 | GCTGAAGGCCGGCAAGACA |
| PLVT750 | GGGATTCCACTCGTGTGTT |
| PLVT751 | TCTCAGATTTCTGAGGAAA |
| CCN2 |  |
| VshRNA1 | ATGTCAAACAAATAGTCTATC |
| VshRNA2 | CATCTTTGAATCGCTGTACTA |
| VshRNA3 | GCATGAAGACATACCGAGCTA |
